# Discovery and Characterization of the Naturally Occurring Inhibitors Against Human Pancreatic Lipase in *Ampelopsis grossedentata*

**DOI:** 10.3389/fnut.2022.844195

**Published:** 2022-02-25

**Authors:** Xiao-Ya Qin, Xu-Dong Hou, Guang-Hao Zhu, Yuan Xiong, Yun-Qing Song, Liang Zhu, Dong-Fang Zhao, Shou-Ning Jia, Jie Hou, Hui Tang, Guang-Bo Ge

**Affiliations:** ^1^Key Laboratory of Xinjiang Phytomedicine Resource and Utilization, Ministry of Education, Pharmacy School of Shihezi University, Xinjiang, China; ^2^Shanghai Frontiers Science Center of TCM Chemical Biology, Institute of Interdisciplinary Integrative Medicine Research, Shanghai University of Traditional Chinese Medicine, Shanghai, China; ^3^College of Basic Medical Sciences, Dalian Medical University, Dalian, China; ^4^Qinghai Hospital of Traditional Chinese Medicine, Xining, China

**Keywords:** human pancreatic lipase (hPL), obesity, *Ampelopsis grossedentata*, myricetin, inhibition mechanism

## Abstract

Pancreatic lipase (PL) inhibitor therapy has been validated as an efficacious way for preventing and treating obesity and overweight. In the past few decades, porcine PL (pPL) is widely used as the enzyme source for screening the PL inhibitors, which generates a wide range of pPL inhibitors. By contrast, the efficacious inhibitors against human PL (hPL) are rarely reported. This study aims to discover the naturally occurring hPL inhibitors from edible herbal medicines (HMs) and to characterize the inhibitory mechanisms of the newly identified hPL inhibitors. Following the screening of the inhibition potentials of more than 100 HMs against hPL, *Ampelopsis grossedentata* extract (AGE) displayed the most potent hPL inhibition activity. After that, the major constituents in AGE were identified and purified, while their anti-hPL effects were assayed *in vitro*. The results clearly showed that two abundant constituents in AGE (dihydromyricetin and iso-dihydromyricetin) were moderate hPL inhibitors, while myricetin and quercetin were strong hPL inhibitors [half-maximal inhibitory concentration (*IC*_50_) values were around 1.5 μM]. Inhibition kinetic analyses demonstrated that myricetin and quercetin potently inhibited hPL-catalyzed near-infrared fluorogenic substrate of human pancreatic lipase (DDAO-ol) hydrolysis in a non-competitive inhibition manner, with *K*_*i*_ values of 2.04 and 2.33 μM, respectively. Molecular dynamics simulations indicated that myricetin and quercetin could stably bind on an allosteric site of hPL. Collectively, this study reveals the key anti-obesity constituents in AGE and elucidates their inhibitory mechanisms against hPL, which offers convincing evidence to support the anti-obesity and lipid-lowering effects of this edible herb.

## Introduction

Obesity and obesity-associated metabolic disorders are serious and global public health problems (1, 2). Although many factors (including genetic, physiological, medicinal, and behavioral factors) participate in the etiology of obesity (3), the most common reason leading to this metabolic disorder is a long-term imbalance between food intake and energy expenditure (4, 5). Over the past 50 years, accompanied by the increase in high-fat and high-sugar diets, as well as insufficient physical activity (6, 7), ~1.9 billion adults in the world were overweight and at least 650 million of them were obese (8, 9). More startlingly, the prevalence of overweight and obesity among children and adolescents has risen dramatically from just 4% in 1975 to over 18% in 2016 (10). The prevalence of obesity is strongly associated with a variety of metabolic disorders, such as hypertension, cardiovascular diseases, non-alcoholic liver disease, type II diabetes, stroke, and various types of cancer (11–13). Consequently, reducing the morbidity related to obesity has become a public health priority. Over the past few decades, several feasible strategies have been proposed for preventing and treating obesity, such as blocking lipid absorption, accelerating lipid metabolism, regulating lipid accumulation, and lipid signal transduction, as well as gastrectomy (14). Among all reported strategies for preventing and treating obesity, blocking lipid absorption by using food or herbal products to lose weight is very popular for obese patients (15, 16).

Pancreatic lipase (PL), a key digestive enzyme responsible for the hydrolysis of dietary triglycerides in the gastrointestinal tract, has gained considerable attention as an important anti-obesity target (17). In the past few decades, a variety of edible herbal medicines (HMs) and their constituents have been reported with anti-PL effects (18–20). However, in most studies, porcine PL (pPL) is frequently used as the enzyme source for screening and characterizing PL inhibitors, which generates a wide range of pPL inhibitors (21–24). By contrast, the efficacious inhibitors against human PL (hPL) are rarely reported. Although the amino acid sequence identity between the pPL and hPL was relatively high (~86%), the large species differences in inhibitor response have been reported (25–27). Thus, it is urgent and necessary to find more efficacious hPL inhibitors as anti-obesity agents by using hPL as the enzyme source. Ideal orally administrated hPL inhibitors for preventing and treating obesity or obesity-associated complications should meet the following three requirements, good safety profile (28), high efficacy (29), high local exposure to the gastrointestinal tract, and very low exposure into the circulatory system which could minimize the influence on the endogenous metabolism in the liver or other deep organs (30, 31). To the best of our knowledge, the ideal orally administrated anti-obesity products *via* inhibiting hPL that meet the above-mentioned requirements are rarely reported. Therefore, it is urgent and necessary to discover more efficacious anti-PL agents or products with good safety profiles for preventing and treating obesity-associated complications.

Recently, a fluorescence-based high-throughput assay for screening hPL inhibitors was established by us in which hPL was used as the enzyme source and a near-infrared fluorogenic substrate (DDAO-ol) was used as probe substrate (32). With the help of this newly developed hPL inhibition assay, more than one hundred HMs extracts were collected and their anti-hPL activities were assayed. The preliminary screening results clearly showed that an edible herbal product, *A. grossedentata* extract (AGE), displayed the most potent anti-hPL effect. *A. grossedentata* has traditionally been used as a health tea and folk HM by ethnic minorities in China and displayed a wide variety of beneficial properties, such as clearing away heat, detoxification, activating blood circulation, and dissipating blood stasis (33). In this case, the bioactive substances in AGE and their anti-hPL mechanisms were carefully investigated by using a panel of techniques, such as liquid chromatography-time of flight tandem mass spectrometer (LC–TOF–MS/MS)-based chemical profiling, reversed-phase liquid chromatographic separation, hPL inhibition assay, and molecular dynamics simulations. This study aims to reveal the key anti-obesity constituents in AGE and elucidate their inhibitory mechanisms against hPL, which will offer direct evidence to support the anti-obesity and lipid-lowering effects of AGE.

## Materials and Methods

### Chemicals and Materials

*A. grossedentata* extract was provided by Eastsign Foods Co., Ltd. (Quzhou, China), other HMs were provided by Tianjiang Pharmaceutical Co., Ltd (Jiangsu, China). Dihydromyricetin, iso-dihydromyricetin, myricitrin, and myricetin were isolated by reverse-phase liquid chromatography. Taxifolin was purchased from Sichuan Weikeqi Biotechnology. Quercetin was purchased from the Shanghai Standard Technology Co., Ltd (Shanghai, China). Reynoutrin was obtained from the BioBioPha Co., Ltd. The positive inhibitors (sanggenone C and orlistat) were purchased from the Dalian Meilun Biotech Co., Ltd (Dalian, China). The purity of all compounds was higher than 98%. The stock solutions of each inhibitor and positive inhibitors were solubilized in grade dimethyl sulfoxide (DMSO, Tedia, USA). The specific substrate (DDAO-ol) and its hydrolytic product (DDAO) were obtained from the previous study (32). Expression and purification of hPL were performed according to the previous study (32). Porcine bile salt was obtained from the Dalian Meilun Biotech Co., Ltd (Dalian, China). Tris was purchased from Roche Diagnostics GmbH (Mannheim, Germany). Tris-HCl buffer (pH 7.4, 25 mM, 150 mM NaCl, 1 mM CaCl_2_) was prepared by using Milli-Q Water (Millipore, USA). LC grade formic acid, acetonitrile, and methanol were purchased from Tedia Company (Fairfield, USA). All the other chemicals, such as NaCl and CaCl_2_, were of analytical grade and obtained from the Sinopharm Chemical Reagent Co., Ltd (Shanghai, China).

### PL Inhibition Assay

The inhibitory activity of each inhibitor against hPL was measured by using DDAO-ol as substrate, while orlistat and sanggenone C were used as positive inhibitors against hPL. Briefly, the incubation mixtures (100 μl, total volume) contained 66 μl Tris-HCl buffer, 10 μl hPL solution (0.5 μg/ml, final concentration), 20 μl porcine bile salt (0.1 mg/ml, final concentration), and 2 μl each inhibitor. After pre-incubating at 37°C for 3 min, the substrate of DDAO-ol (20 μM, final concentration) was added to the initial reaction. Subsequently, the fluorescent signals of the hydrolytic product of DDAO-ol (DDAO) were quantified by using the microplate reader (Spectra Max M4, Austria), with the wavelength of 600 and 660 nm for excitation and emission, respectively. The standard curve was plotted using DDAO as product standard (Supplementary Figure S6). The control incubations without inhibitors were also performed.

### Chemical Profiling of AGE by Liquid Chromatography–Time of Flight Tandem Mass Spectrometer

To identify and characterize the naturally occurring hPL inhibitors in AGE, a UFLC system (Kyoto, Japan) coupling with a Triple TOF 5600 Mass Spectrometer system (Foster City, CA, USA) was performed to analyze predominant constituents. The chromatographic separation was employed by the Shimadzu VP-ODS C_18_ column (2.0 mm × 250 mm, 4.6 μm) with a flow rate of 0.4 ml/min. The mobile phase was made up of water (0.1% formic acid) (A) and acetonitrile (B). The mobile phase program was shown in Supplementary Table S1. The column temperature was set at 40°C. The AGE sample (10 mg/ml) injection volume was 3 μl. The constituents were further analyzed *via* LC–TOF–MS/MS equipped with an electrospray ionization (ESI) source in negative ion mode. The MS parameters settings were shown in Supplementary Table S2.

### Inhibition Kinetic Analyses

To investigate the inhibition kinetic types and to determine the inhibition constants (*K*_*i*_) of two flavonoids (such as myricetin and quercetin) against hPL, the inhibition kinetics were carefully investigated *via* performing a series of kinetic analyses (34–36). Briefly, increasing concentrations of each inhibitor were mixed with hPL in the above-mentioned incubation system, while the reactions were started by adding DDAO-ol (at various concentrations) following 3 min pre-incubation at 37°C. The inhibition constants (*K*_*i*_) were calculated as depicted in previous studies (37, 38).

### Molecular Dynamic Simulations

To explore the allosteric mechanism of the newly identified inhibitors against hPL, docking simulations and molecular dynamic (MD) simulations were conducted. Prior to MD simulations, each ligand was emplaced into the allosteric pocket of hPL that was predicted by Cavityplus (39). The lipase–colipase complex structure of hPL (PDB code: 1LPA) was acquired from Protein Date Bank (https://www.rcsb.org/) (40, 41). First, the coordinate files of macromolecule (hPL) and each ligand (myricetin and quercetin) were ameliorated by AutoDockTools 1.5.6, such as only retaining hydrogen atoms of polarity, calculating atomic electric charges, and defining AD4 atom types (42). Second, the allosteric pocket of hPL was defined as the ligand-binding sites for each ligand. Then, top docking poses computed by Autodock Vina were collected for the following MD simulations.

The MD simulations of hPL-allosteric agent complexes were operated by GROMACS (43). Systems of receptor–ligand topology were constructed for simulations. In the previous step of MD simulations, a 50,000-step steepest descent energy minimization was operated. ApohPL and hPL-ligand atom coordinates were solvated with the rigid water model that has a Lennard-Jones site on the Oxygen and bare charge sites on the Hydrogens (TIP3P) water model, and the system was neutralized by sodium or chlorine ions. Next, the system was balanced by 100-ps constant volume and temperature (NVT) heating to 310 K and 100-ps performing the simulation with constant pressure and temperature (NPT). The initial energy-minimized balanced topology of systems was subjected to 100 ns MD at 310 K (V-rescale thermostat) and 1 bar (Parrinello–Rahman barostat). To go a step further on the allosteric binding modes between hPL and inhibitors, equilibrium conformations were clustered, and the largest center structure was kept for ligand–receptor interaction analysis *via* Discovery Studio Visualizer (BIOVIA Discovery Studio 2019, Dassault Systèmes, San Diego, USA).

### Statistical Analysis

All inhibition assays were performed in triplicate, while the data were expressed as mean ± SD. *IC*_50_ values and *K*_*i*_ values were determined by GraphPad Prism 8.0 (GraphPad Software, Inc., La Jolla, USA).

## Results

### Discovery of the HMs With Strong hPL Inhibition Activity

**Figure 1:**
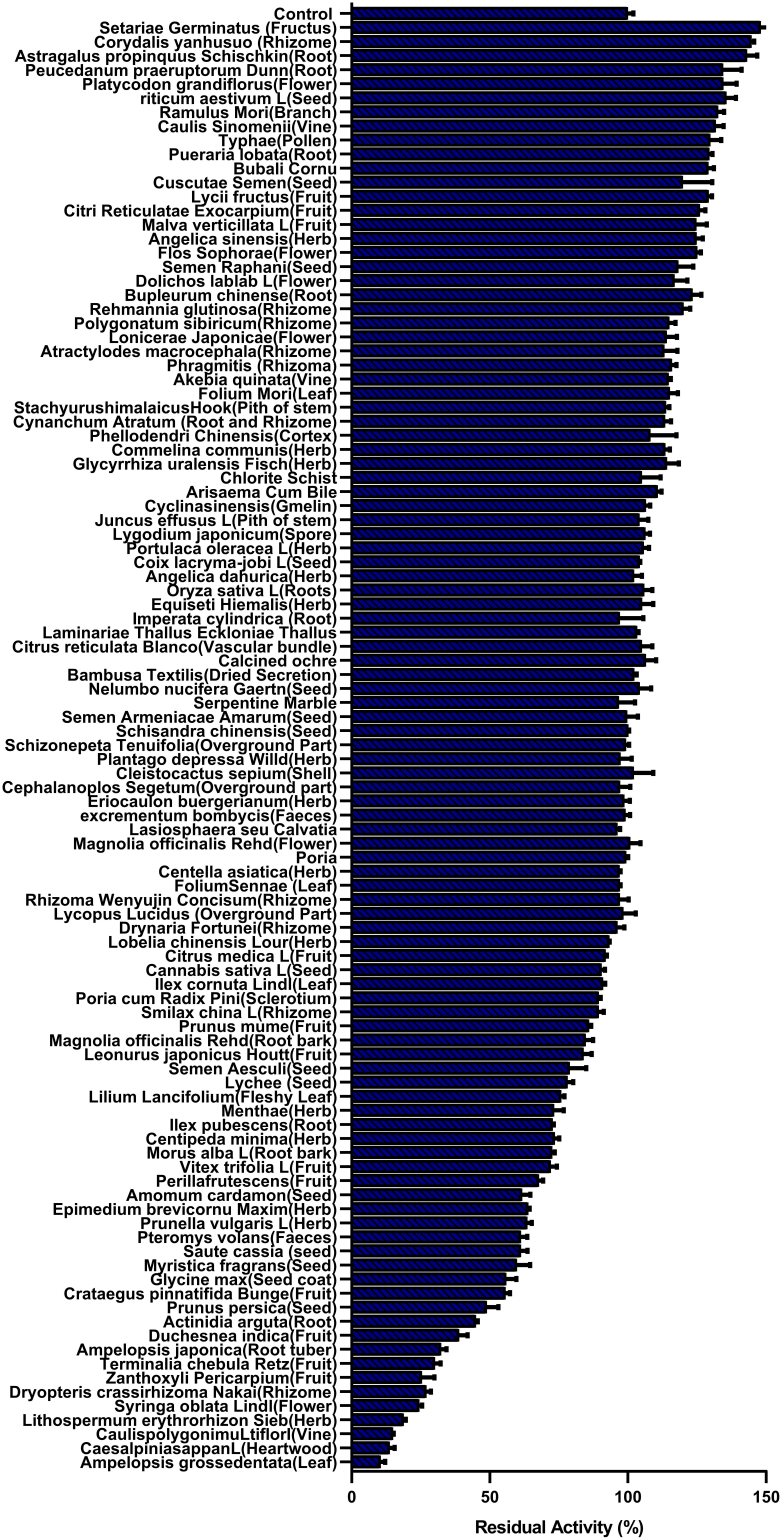
The residual activity of human pancreatic lipase (hPL) in the presence of all tested herbal medicines (100 μg/ml, final concentration) was measured by catalyzed DDAO-ol hydrolysis. Data were shown as mean ± SD.

First, the hPL inhibitory activities of more than 100 HMs were assayed under identical conditions by using a high dosage (100 μg/ml, final concentration) (44, 45). As shown in Figure 1, although most of the tested HMs displayed weak anti-hPL effects, AGE showed the most potent anti-hPL effect. Next, to quantitatively characterize the inhibitory effects of AGE against hPL, the dose-inhibition curve of AGE against hPL-catalyzed DDAO-ol hydrolysis was plotted using increasing concentrations of AGE. As shown in Supplementary Figure S1, AGE dose-dependently inhibited hPL-catalyzed DDAO-ol hydrolysis in recombinant hPL, with a calculated *IC*_50_ value of 6.15 ± 0.71 μg/ml. Meanwhile, the time-dependent inhibition assay showed that the hPL inhibition potential of AGE was not changed following long pre-incubation time (Supplementary Figure S2), suggesting that the constituents in AGE were not time-dependent inhibitors of hPL. These results demonstrate that AGE inhibits hPL-catalyzed DDAO-ol hydrolysis *via* a dose-dependent and reversible manner, implying that this HM contains naturally occurring hPL inhibitor(s).

### Chemical Profiling and Isolation of the Major Constituents in AGE

**Figure 2:**
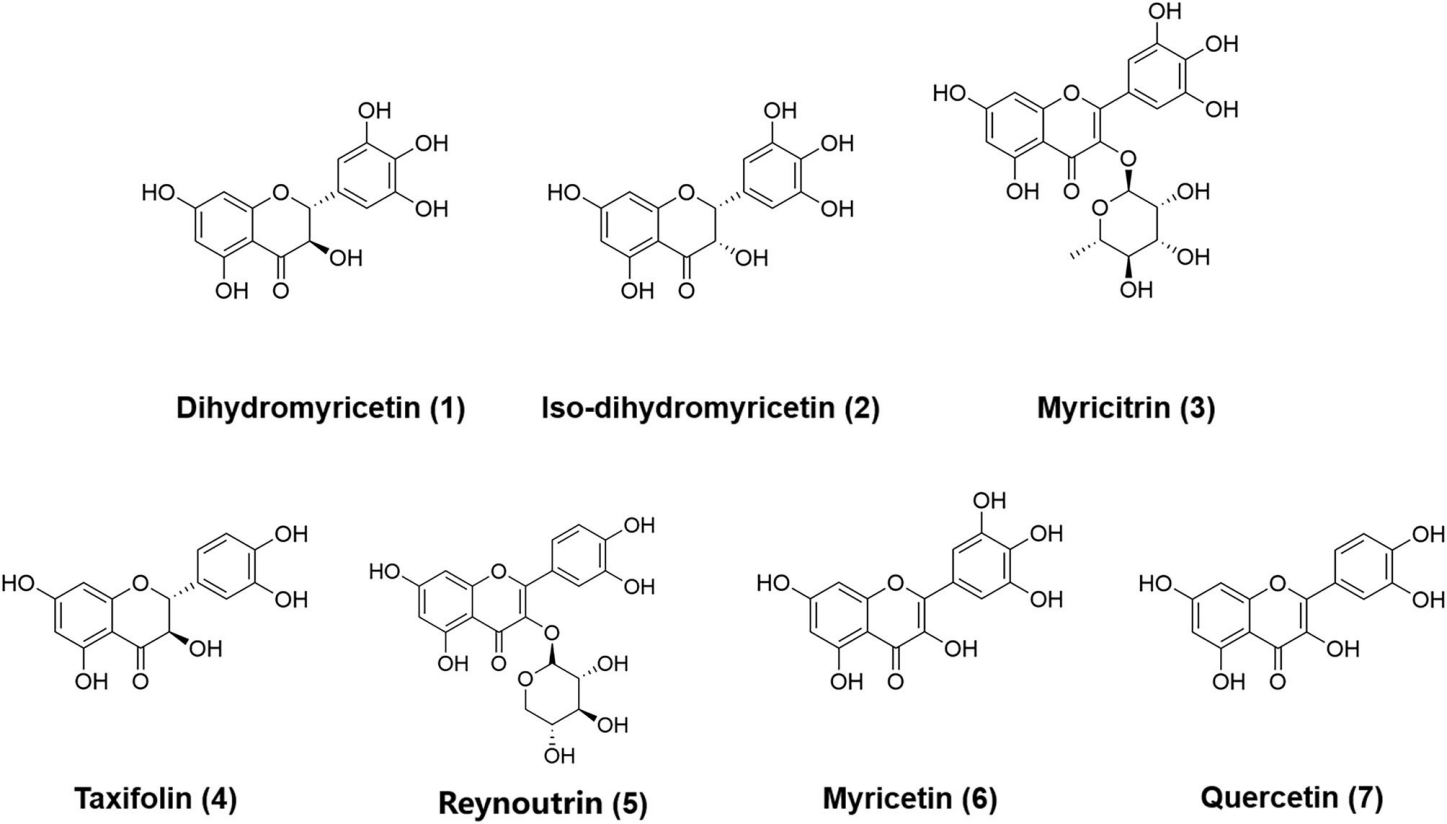
The chemical structures of dihydromyricetin 1, iso-dihydromyricetin 2, myricitrin 3, taxifolin 4, reynoutrin 5, myricetin 6, and quercetin 7.

Next, the chemical constituents in AGE were characterized by a reliable LC–TOF–MS/MS method under the negative ion mode. As shown in Supplementary Figure S3, a total of nine major constituents in AGE, including 3-dihydroxyquercetin, dihydromyricetin, iso-dihydromyricetin, myricitrin, taxifolin, reynoutrin, quercetin-3-*O*-α-L-rhamnopyranoside, myricetin, and quercetin, were tentatively identified and their fragment ions were carefully characterized (33). The detailed fragment ions of nine identified constituents were listed in Table 1 and Supplementary Figures S7–S15. Four abundant constituents in AGE, including dihydromyricetin, iso-dihydromyricetin, myricitrin, and myricetin, were purified by reverse-phase liquid chromatography and fully characterized by ^1^H NMR and ^13^C nuclear magnetic resonance (NMR) spectra (Supplementary Figures S16–S19). Meanwhile, taxifolin, reynoutrin, and quercetin were confirmed by comparing their retention times on reverse-phase liquid chromatography, UV spectra, and MS/MS spectra with that of authentic standards. The chemical structures of the identified flavonoids in AGE were shown in Figure 2.

**Table 1:** Identification of the main constituents in *Ampelopsis grossedentata* extract (AGE) by using LC–TOF–MS/MS.

**No**.	**RT (min)**	**Pseudo-** **molecular ion**	**m/z observed**	**Formula**	**Identification**	**MS/MS spectra**
1.	6.959	[M-H]^−^	319.0461	C_15_H_12_O_8_	3-dihydroxyquercetin	319.0503,301.0357,257.0469,233.0463, 215.0355,193.0147
2.	10.611	[M-H]^−^	319.0462	C_15_H_12_O_8_	Dihydromyricetin	319.0476,301.0366,257.0465, 215.0357,193.0152,175.0044
3.	11.413	[M-H]^−^	319.0462	C_15_H_12_O_8_	Iso-dihydromyricetin	319.0473,301.0370,257.0464, 233.0470,215.0358,193.0149
4.	12.894	[M-H]^−^	463.0884	C_21_H_20_O_12_	Myricitrin	463.0884,316.0229,287.0204,271.0254
5.	13.464	[M-H]^−^	303.0515	C_15_H_12_O_7_	Taxifolin	303.0527,285.0417,275.0579,259.0631, 217.0520,199.0410,175.0414,151.0040
6.	14.004	[M-H]^−^	433.0774	C_20_H_18_O_11_	Reynoutirn	433.0772,300.0290,271.0259,255.0304
7.	14.571	[M-H]^−^	447.0938	C_21_H_20_O_11_	Quercetin-3-*O*-α-L-rhamnopyranoside	447.0949,300.0293,271.0265,255.0316, 243.0314
8.	15.521	[M-H]^−^	317.031	C_15_H_10_O_8_	Myricetin	317.0337,299.0221,289.0381227.0363, 179.0014,151.0053,137.0260,109.0304
9.	18.091	[M-H]^−^	301.0359	C_15_H_10_O_7_	Quercetin	301.0392,273.0429,179.0008, 151.0056,121.0307,107.0147

### The Anti-hPL Effects of the Major Constituents in AGE

**Figure 3:**
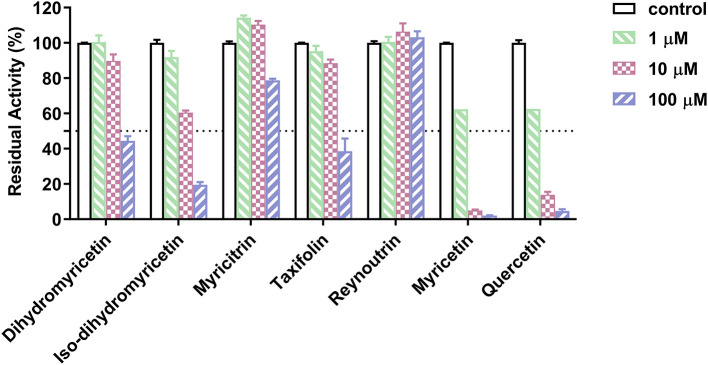
Inhibitory effects of seven major constituents in the *Ampelopsis grossedentata* extract (AGE) against hPL-catalyzed DDAO-ol hydrolysis. Data were shown as mean ± SD.

**Figure 4:**
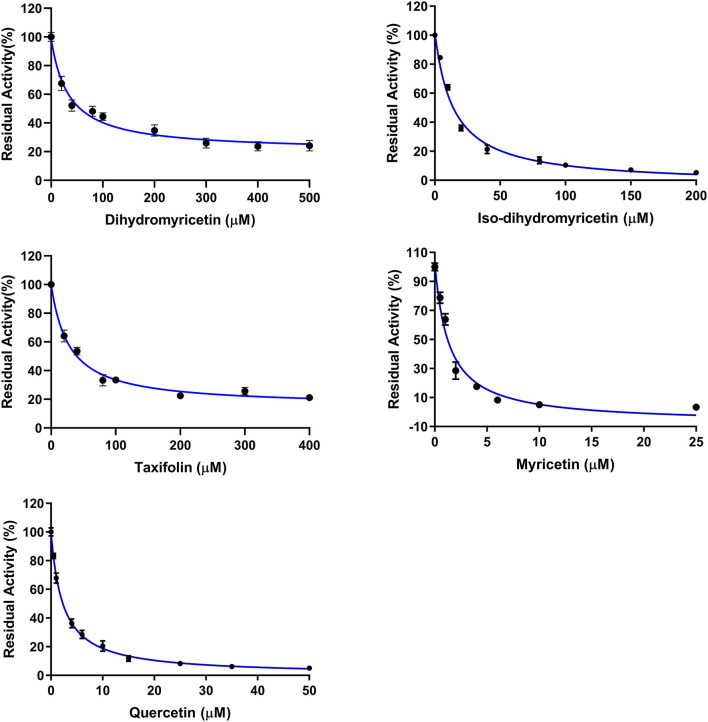
Dose-inhibition curves of dihydromyricetin, iso-dihydromyricetin, taxifolin, myricetin, and quercetin against hPL-catalyzed DDAO-ol hydrolysis. Data were shown as mean ± SD.

Subsequently, the anti-hPL effects of seven major constituents in AGE were tested at three inhibitor concentrations (1, 10, and 100 μM). The results showed that myricetin and quercetin displayed potent anti-hPL effects (Figure 3), with the residual activities of <15% at 10 μM. By contrast, dihydromyricetin, iso-dihydromyricetin, taxifolin displayed moderate hPL inhibition activities (Figure 3), while myricitrin and reynoutrin did not inhibit hPL even at high dose (100 μM). Further investigations showed that myricetin and quercetin (46) dose-dependently inhibited hPL-catalyzed DDAO-ol hydrolysis, with the *IC*_50_ values of 1.34 ± 0.18 μM, 1.53 ± 0.10 μM, respectively (Figure 4, Table 2). Notably, the anti-hPL effects of these two flavonoids isolated from AGE are more potent than that of Sanggenone C (*IC*_50_ = 3.46 μM), a previously reported naturally occurring reversible hPL inhibitor. Meanwhile, the *IC*_50_ values of dihydromyricetin, iso-dihydromyricetin, and taxifolin (47) were also determined as 34.28 ± 4.63 μM, 14.37 ± 1.21 μM, and 27.83 ± 2.92 μM, respectively (Figure 4, Table 2). These results clearly demonstrate that five flavonoids from AGE are naturally occurring hPL inhibitors, while two flavonoids (myricetin and quercetin) display strong hPL inhibition activity, which encourages us to further investigate the inhibition kinetics and inhibitory mechanisms of these two flavonoids.

**Table 2:** The contents, *IC*_50_ values, *K*_*i*_ values, and the inhibition modes of the bioactive constituents in AGE against human pancreatic lipase (hPL).

**Compound**	**MW**	**Content (μg/mg)**	**IC_**50**_ (μM)**	***K_i_*** **(μM)**	**Inhibition mode**	**Goodness** **of fit (***R***^2^)**
Myricetin	318.24	8.22	1.34 ± 0.18	2.04 ± 0.75	Non-competitive	0.99
Quercetin	302.24	–	1.53 ± 0.10	2.33 ± 0.71	Non-competitive	0.99
Iso-dihydromyricetin	320.25	45.65	14.37 ± 1.21	–	–	–
Dihydromyricetin	320.25	88.15	34.28 ± 4.63	–	–	–
Taxifolin	304.25	4.81	27.83 ± 2.92	–	–	–
Myricitrin	464.38	–	>100	–	–	–
Reynoutirn	434.35	–	>100	–	–	–
SanggenoneC^a^	708.71	–	3.46 ± 0.07	–	–	–
Orlistat^a^	495.73	–	6.16 ± 0.22 nM	–	–	–

a *Sanggenone C and orlistat were used as positive inhibitors of hPL*.

### Determination of the Contents of Major Constituents in AGE With hPL Inhibition Activity

Subsequently, the contents of four major constituents (including dihydromyricetin, iso-dihydromyricetin, taxifolin, and myricetin) in AGE with hPL inhibition activity were determined by LC–UV. Prior to quantitative determination, the standard curves of these four ingredients in AGE were plotted and depicted in Supplementary Figure S20. After that, the contents of dihydromyricetin, iso-dihydromyricetin, taxifolin, and myricetin in AGE were determined as 88.15 μg/mg, 45.65 μg/mg, 4.81 μg/mg, and 8.22 μg/mg, respectively (as listed in Table 2). These findings suggest that the contents of dihydromyricetin and iso-dihydromyricetin in AGE are extremely high, while the contents of taxifolin and myricetin are at a moderate level (ranging from 4.81 to 8.22 μg/mg).

### The Inhibitory Behaviors of Two Potent hPL Inhibitors in AGE

**Figure 5:**
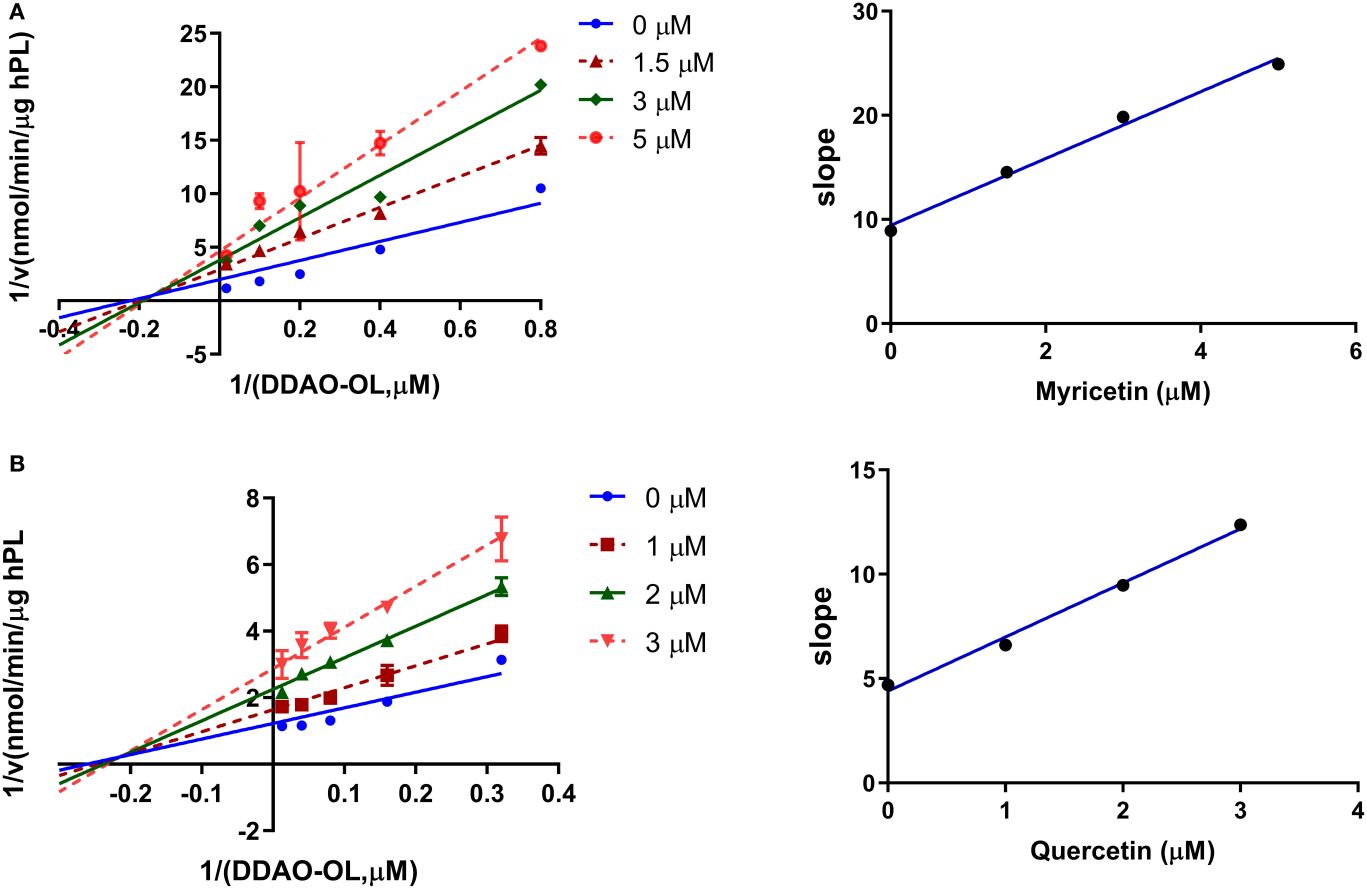
Inhibition kinetics of myricetin **(A)**, and quercetin **(B)** toward the activity of hPL. Left, Lineweaver–Burk plots for each compound against hPL-catalyzed DDAO-ol hydrolysis. Right, the second plot of slopes from the Lineweaver–Burk plots.

Next, the inhibition kinetics and the inhibition constants (*K*_*i*_) of two flavonoids (myricetin and quercetin) with strong hPL inhibition activity were investigated. Prior to inhibition kinetic analyses, the time-dependent inhibition assays of myricetin and quercetin were performed. As shown in Supplementary Figure S2, the anti-hPL effects of myricetin and quercetin were not time-dependent, suggesting that these two flavonoids isolated from AGE were reversible hPL inhibitors rather than time-dependent inactivators. After that, the inhibition kinetics of myricetin and quercetin against hPL-catalyzed DDAO-ol hydrolysis were carefully characterized. The Lineweaver–Burk plots demonstrated that myricetin and quercetin potently inhibited the hPL in a non-competitive manner (Figure 5), with the apparent *K*_*i*_ values of 2.04 ± 0.75 μM and 2.33 ± 0.71 μM, respectively (Table 2). These observations clearly demonstrate that myricetin and quercetin strongly inhibit hPL in a non-competitive inhibition mode, with the *K*_*i*_ values of less than 2.5 μM.

### Molecular Dynamic Simulations

**Figure 6:**
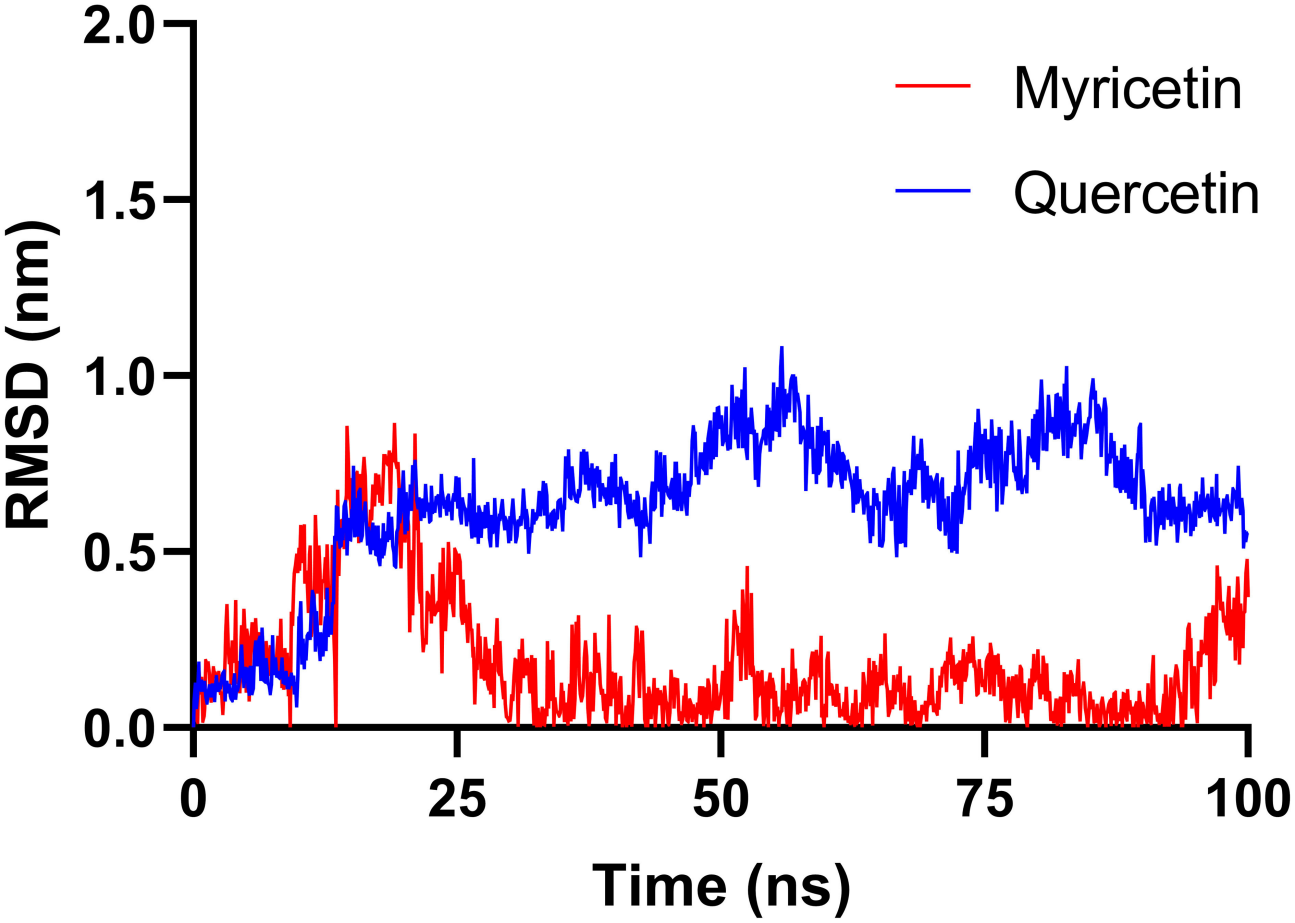
The Root Mean Square Deviation (RMSD) fluctuation of hPL and complex bound with myricetin and quercetin.

**Figure 7:**
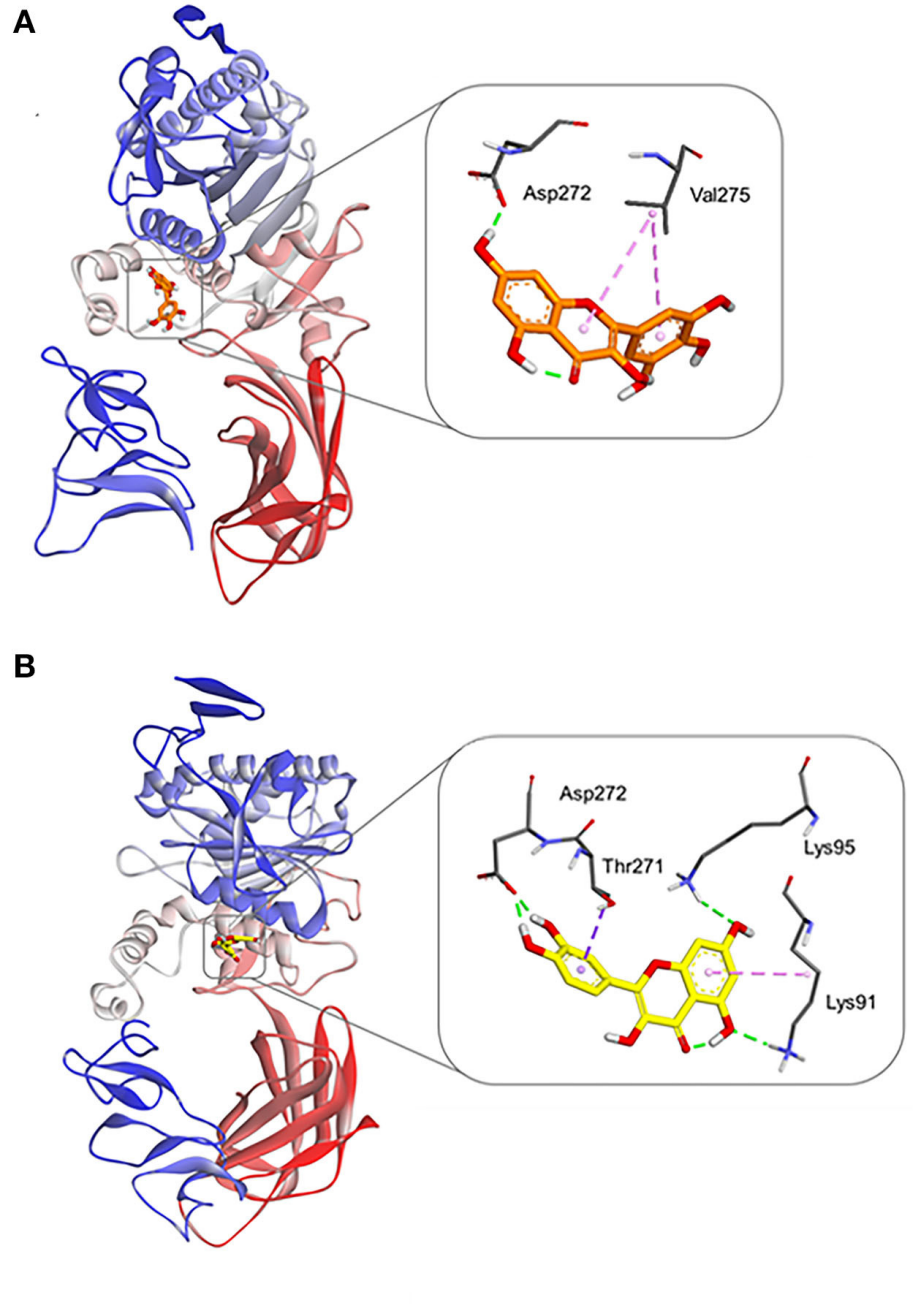
Molecular docking results of myricetin and quercetin into the crystal structure of hPL (PDB ID: 1LPA) by Autodock Vina. Equilibrium conformations of myricetin **(A)**, and quercetin **(B)** in stereo overview and detailed view.

Finally, MD simulations were carefully conducted to explore the binding poses of myricetin and quercetin on hPL. Each ligand was firstly docked into a predicted allosteric site of hPL (Supplementary Figure S21), and then MD simulations of hPL-ligand systems were operated as described in Section **Molecular Dynamic Simulations**. As shown in Figure 6, following docked with myricetin or quercetin, the gyro-radius of hPL could be remarkably stabilized. Notably, these ligands could form a strong hydrogen bonding interaction with Asp272, a key residue right below the catalytic pocket of hPL (Figure 7). Meanwhile, the binding of quercetin caused a shift on the “flap” of hPL, as depicted in Supplementary Figure S22. It has been reported that the flap played a significant role in catalytic activity, whose designated motion makes the catalytic residues accessible to the substrate (48). Such irregular conformational change could be mortal to the hydrolytic activity of hPL. Apart from the H-bonding interactions between Asp272 and these two inhibitors, myricetin formed Pi-alkyl interaction with Val 275, while quercetin formed complex hydrophobic interactions (Pi-alkyl and Pi-sigma) with a panel of residues in hPL (such as Thr271, Lys95, and Lys91), as depicted in Figure 7 and Supplementary Figures S23, S24. These hydrophobic interactions were mainly directing the conjugated structure of each flavonoid, which was of importance for the binding of these inhibitors. These observations suggest that myricetin and quercetin can bind on hPL firmly in an allosteric way, which is consistent with the inhibition kinetics results that myricetin and quercetin potently inhibit hPL in an allosteric manner.

## Discussion

It is well-known that hPL is one of the most important enzymes responsible for the digestion of dietary lipid (such as triacylglycerols) in the intestinal tract (14, 49), while hPL inhibitor therapy has been validated as an efficacious and cost-effective way for preventing and treating obesity and overweight. Orlistat, a potent and irreversible inhibitor of mammalian PL, has been approved for obesity treatment for over 20 years. However, this anti-PL agent has been found with a variety of adverse effects (gastrointestinal toxicity, pancreatic damage, metabolic system abnormalities, and high cancer risk) following long-term use (50–52). Thus, there is an urgent need for discovering more efficacious hPL inhibitors or for developing more practical anti-obesity products with good safety profiles for combating obesity. In the past few decades, many studies have reported that a range of HMs (such as the extract of *Ginkgo biloba* and *Cortex Mori Radicis*) are beneficial for the treatment of obesity and obesity-related metabolic diseases (53–56). Meanwhile, these HMs have several inherent advantages for weight loss and treating obesity-associated metabolic disorders, including a good safety profile, high anti-PL efficacy, as well as high gastrointestinal exposure but extremely low exposure in the circulation system. However, most previous studies use pPL to replace hPL as the enzyme source for screening and characterizing PL inhibitors, the inhibition potentials of known edible HMs against hPL and related key anti-hPL constituents are poorly investigated.

This study aims to investigate the inhibition potentials of edible HMs against hPL and to discover and characterize the naturally occurring hPL inhibitors from the most potent anti-hPL HM. Among all tested edible HMs, AGE was found with the most potent anti-hPL effect, with the estimated *IC*_50_ value of 6.15 μg/ml. *A. grossedentata* is known as the “king of flavonoids”, while the content of total flavonoids in AGE is up to 45% (57). Herein, a total of nine flavonoids in AGE were identified and characterized by LC–MS/MS-based chemical profiling. Among them, five flavonoids (including dihydromyricetin, iso-dihydromyricetin, taxifolin, myricetin, and quercetin) were identified as the key constituents responsible for hPL inhibition. Although dihydromyricetin and iso-dihydromyricetin (the most abundant constituents) show moderate hPL inhibition activity, the contents of these two flavonoids are very high (88.15 μg/mg for dihydromyricetin and 45.65 μg/mg for iso-dihydromyricetin). It is estimated that the local exposure of these two agents in the gastrointestinal tract may be over 100 μM (206.08 μM for dihydromyricetin and 106.90 μM for iso-dihydromyricetin, *via* calculating the daily dose of each agent divided by the volume of the human gastrointestinal system), following oral administration with AGE at the daily dosage of this herbal product recommended by Chinese Materia Medica (58). In addition, the local exposure of the other flavonoids (such as taxifolin and myricetin) in AGE in the gastrointestinal tract can reach 11.85 and 19.37 μM, respectively, which are all much higher than their *IC*_50_ values (around 1.5 μM). Moreover, although myricitrin and reynoutrin in AGE display weak inhibitory effects against hPL, these two glycosides can be readily hydrolyzed by gut microbial to form myricetin and quercetin in the gastrointestinal tract, which can potently inhibit hPL (59). Thus, it is easily conceivable from these data that drinking *A. grossedentata* could efficiently inhibit hPL and display satisfactory anti-hPL effects *in vivo*, which in turn, reduce lipid absorption and lose body weight. It is also reported that the local exposure of dihydromyricetin (the most abundant constituent in *A. grossedentata*) in the stomach and small intestine is much higher than that in other deep organs (such as heart, liver, spleen, lung, kidney, and brain) following oral administration, suggesting that dihydromyricetin has very poor oral bioavailability and this agent hardly affects the endogenous metabolism in the liver (60, 61).

Since the flavonoids of AGE play an important role in hPL inhibition, the total flavonoids or AGE can be developed as HMs or other commercial products (such as drinking foods) in the future. It is noteworthy that there are significant differences in the flavonoid content from different batches of *A. grossedentata* (62), the possible reasons for the differences in flavonoid content of *A. grossedentata* are presumably due to seasonal and geographic factors, as well as processing schemes. Therefore, to obtain high-quality raw materials of *A. grossedentata* with satisfactory anti-hPL effect, it is necessary to critically analyze the influence of regions, seasons, and processing techniques on the contents of several anti-hPL flavonoids in *A. grossedentata*. Furthermore, a previous study has reported that dihydromyricetin can be converted into iso-dihydromyricetin (63), while the anti-hPL effect of iso-dihydromyricetin is much stronger than that of dihydromyricetin. In the future, the processing scheme of *A. grossedentata* can be intentionally optimized to convert more dihydromyricetin into iso-dihydromyricetin, which will strongly enhance the anti-hPL effect of AGE products.

## Conclusion

In summary, this work reported the discovery of the bioactive anti-hPL substances from AGE, edible folk medicine in South China, and characterized the inhibitory mechanisms of the newly identified anti-hPL substances from this edible herb. Our results clearly demonstrated that almost all identified flavonoids in AGE were naturally occurring hPL inhibitors in which two abundant flavonoids in AGE (dihydromyricetin and iso-dihydromyricetin) were moderate hPL inhibitors, while myricetin and quercetin were identified as potent hPL inhibitors, with the *IC*_50_ values were around 1.5 μM. A panel of inhibition kinetic assays demonstrated that myricetin and quercetin potently inhibited hPL in a non-competitive manner, with the *K*_*i*_ values of <2.5 μM. MD simulations suggested that myricetin and quercetin could tightly bind on hPL at an allosteric site. Collectively, our findings reveal the key anti-obesity constituents in AGE and elucidate their inhibitory mechanisms against hPL, all these data offer solid evidence to support the anti-obesity and the lipid-lowering effects of AGE and suggest that this edible herb can be developed as dietary supplements for preventing and treating obesity or obesity-associated complications.

## Data Availability Statement

The original contributions presented in the study are included in the article/Supplementary Material, further inquiries can be directed to the corresponding authors.

## Author Contributions

X-YQ: methodology, validation, data curation, and writing–original draft. X-DH: validation, data curation, and writing–original draft. G-HZ and YX: methodology and data curation. Y-QS: investigation. LZ: methodology. D-FZ: formal analysis. S-NJ: resources and funding acquisition. JH: writing–review and editing and project administration. HT: conceptualization, supervision, and project administration. G-BG: conceptualization, supervision, writing–review and editing, and project administration. All authors contributed to the article and approved the submitted version.

## Funding

This work was supported by the National Natural Science Foundation of China (82160739, 81922070, 81973286, and 81973393), Innovation Team and Talents Cultivation Program of National Administration of Traditional Chinese Medicine (No. ZYYCXTD-D-202004), Shuguang Program (No. 18SG40) supported by Shanghai Education Development Foundation and Shanghai Municipal Education Commission, the Program for Innovative Leading Talents of Qinghai Province (2018 and 2019), the Key R&D and Transformation Science and Technology Cooperation Project of Qinghai Province (2019-HZ-819).

## Conflict of Interest

The authors declare that the research was conducted in the absence of any commercial or financial relationships that could be construed as a potential conflict of interest.

## Publisher's Note

All claims expressed in this article are solely those of the authors and do not necessarily represent those of their affiliated organizations, or those of the publisher, the editors and the reviewers. Any product that may be evaluated in this article, or claim that may be made by its manufacturer, is not guaranteed or endorsed by the publisher.
