# Supplementary material for: Discovery and Characterization of the Naturally Occurring Inhibitors Against Human Pancreatic Lipase in Ampelopsis grossedentata
Source: Front Nutr. 2022 Feb 25;9:844195. doi: 10.3389/fnut.2022.844195 (PMC8914261; doi:10.3389/fnut.2022.844195)
Supplement: Supplementary file 1 [file Data_Sheet_1.docx]

Supplementary Material


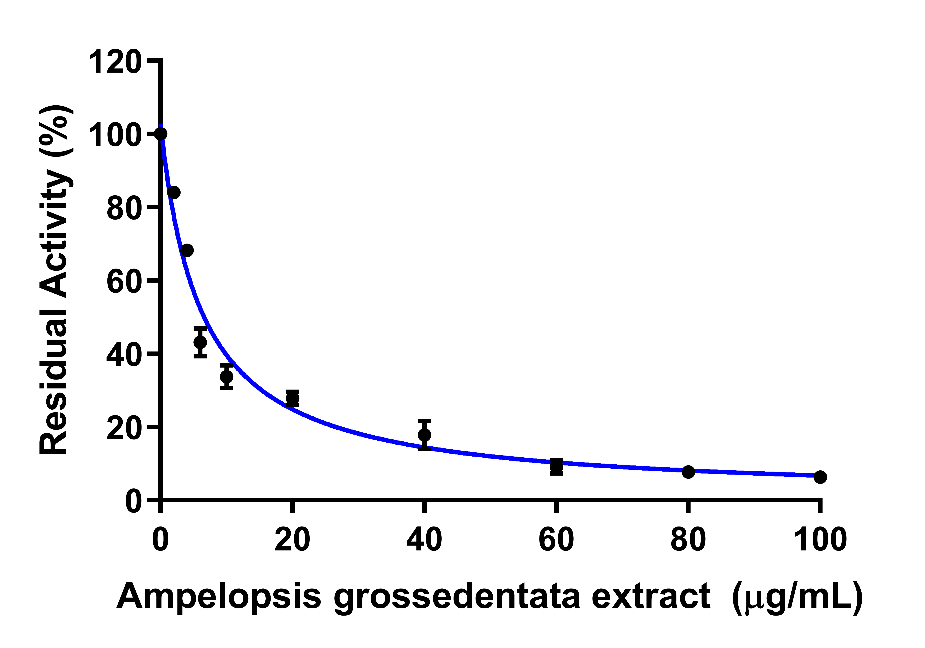


**Fig. S1.** The dose-inhibition curve of hPL-mediated hydrolysis of DDAO-ol by AGE. Data were shown as mean ± SD.


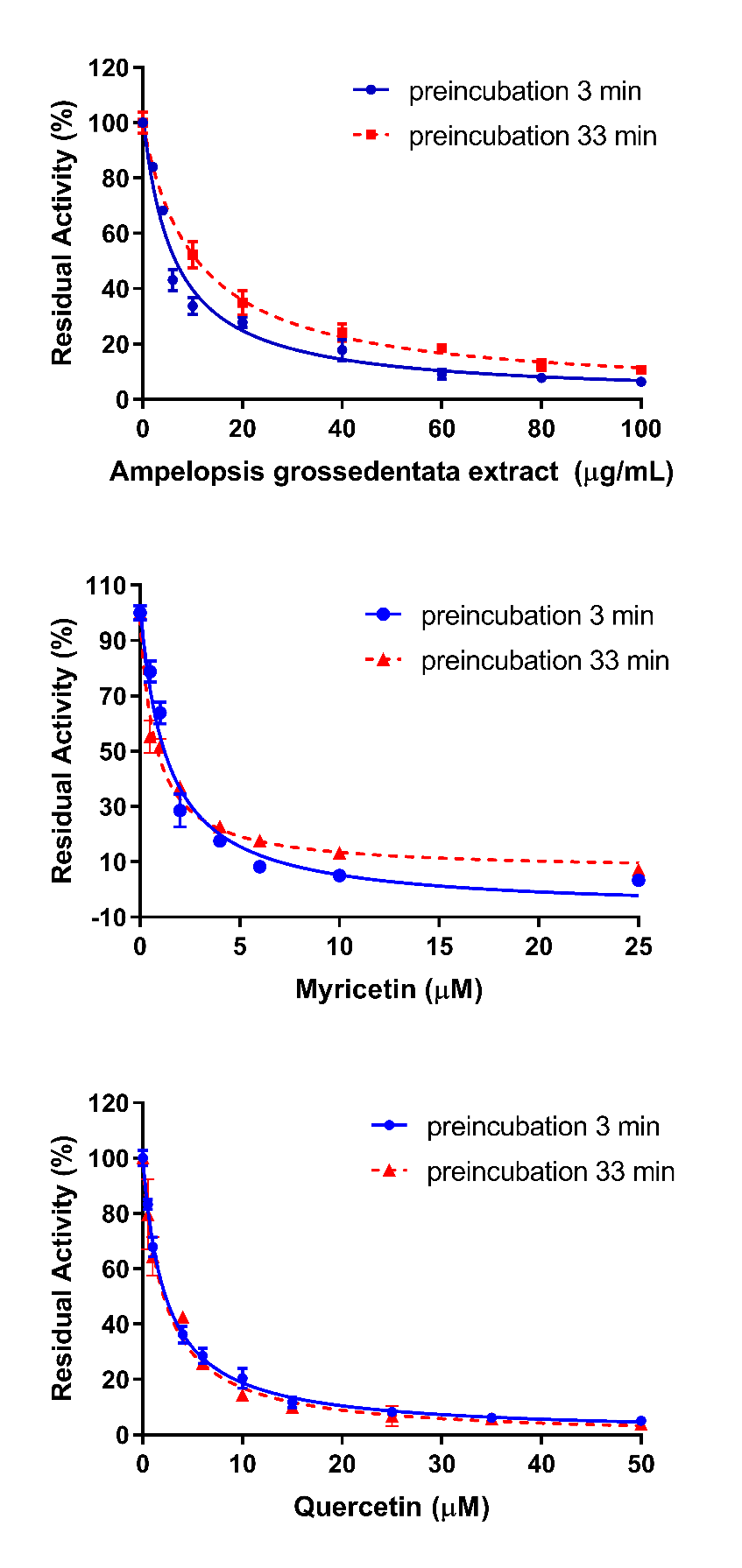


**Fig. S2.** The residual activity of hPL-mediated DDAO-ol hydrolysis by AGE, myricetin and quercetin under different pre-incubation times. Data were shown as mean ± SD.


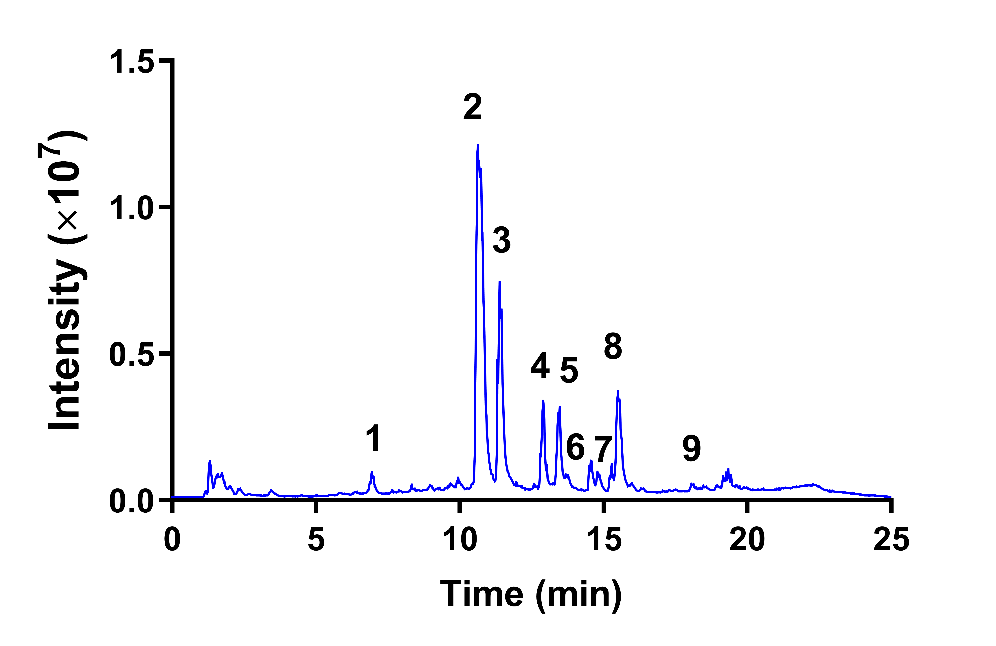


**Fig. S3.** Total ion chromatograph of AGE in negative ion mode.


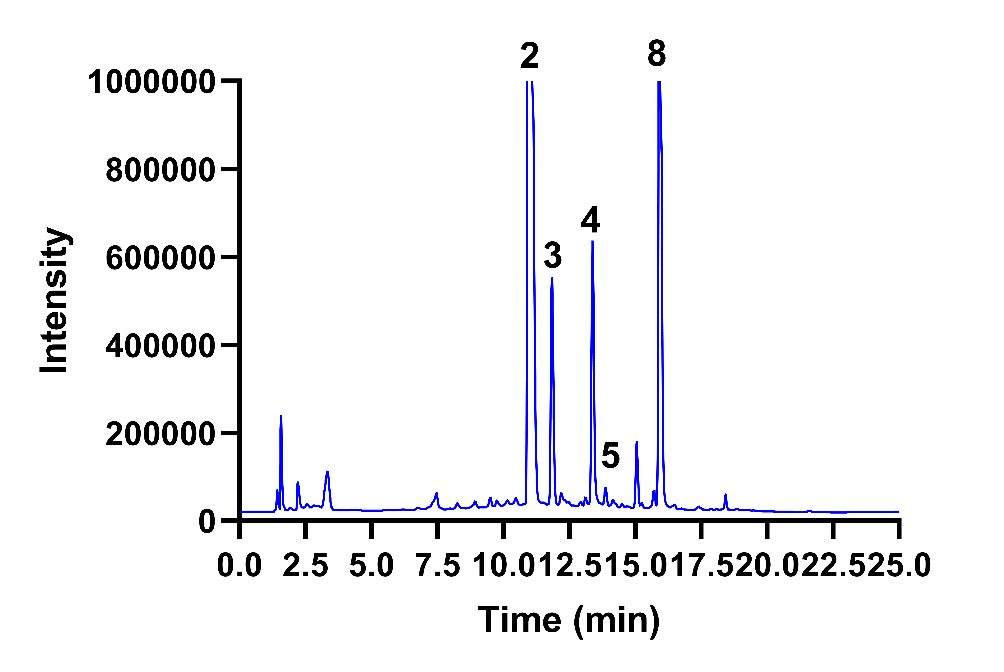


**Fig. S4.** The HPLC-UV chromatogram of AGE at 290 nm.


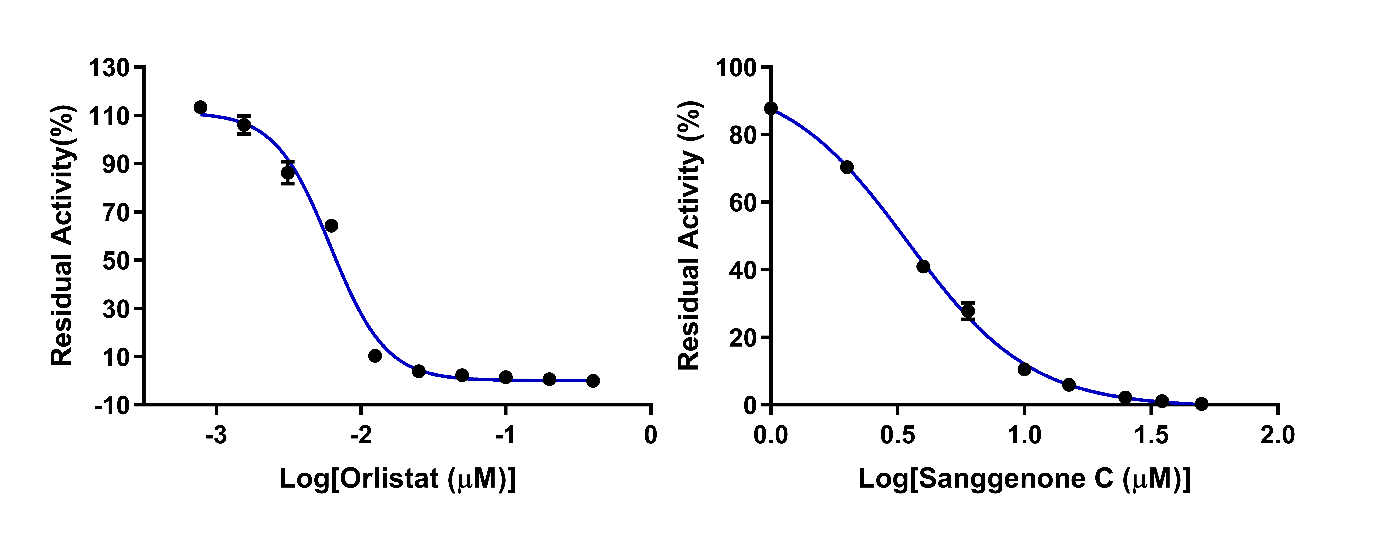


**Fig. S5.** The dose-inhibition curves of orlistat and sanggenone C were determined by hPL-catalyzed DDAO-ol hydrolysis. Data were shown as mean ± SD.


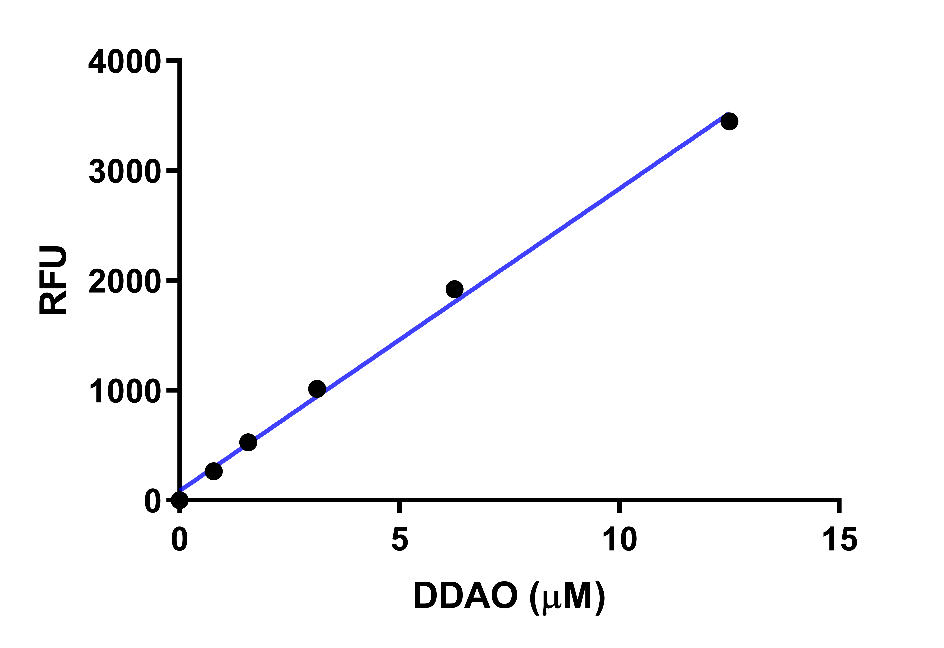


**Fig. S6.** The standard curve of DDAO was determined.


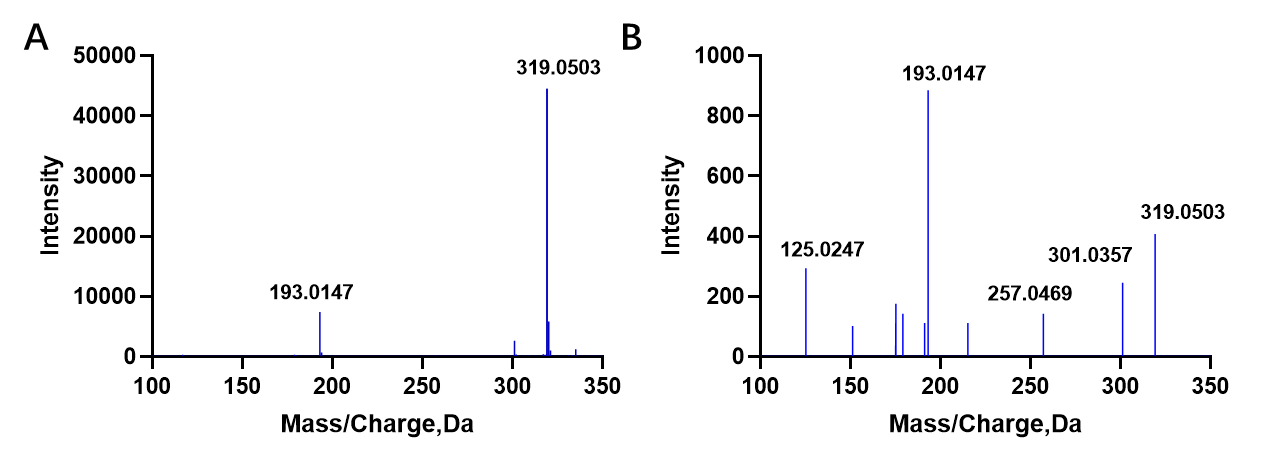


**Fig. S7.** MS^1^ (A) and MS^2^ (B) spectra of 3-dihydroxyquercetin.


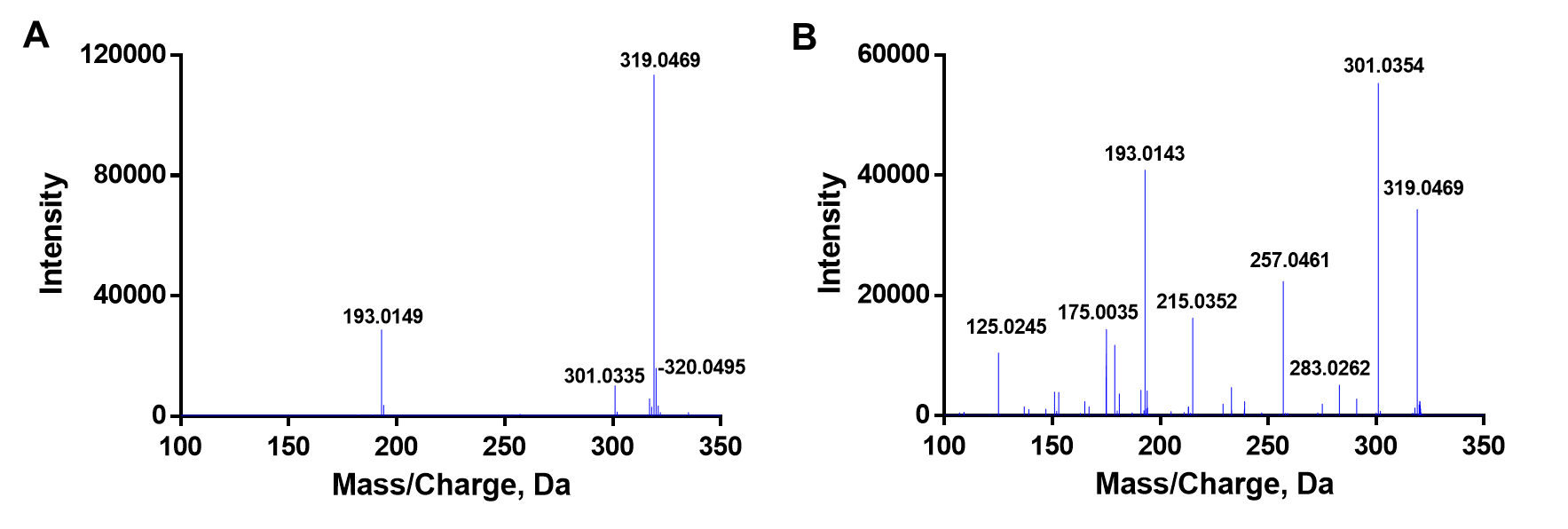


**Fig. S8.** MS^1^ (A) and MS^2^ (B) spectra of dihydromyricetin.


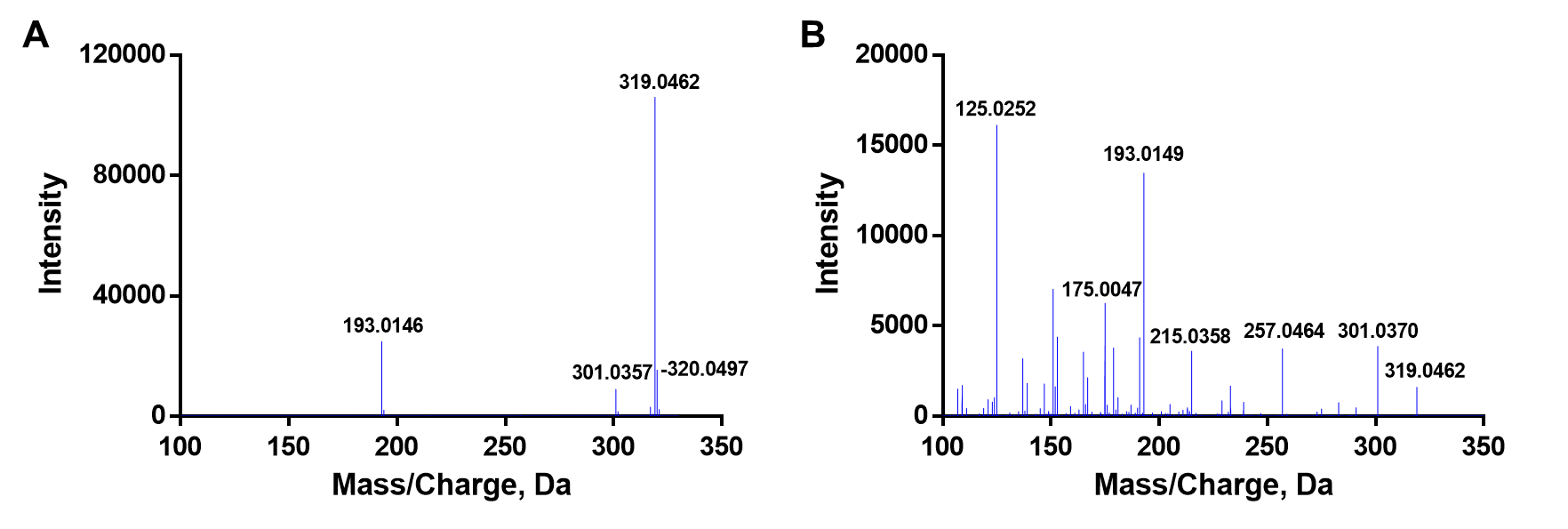


**Fig. S9.** MS^1^ (A) and MS^2^ (B) spectra of iso-dihydromyricetin.


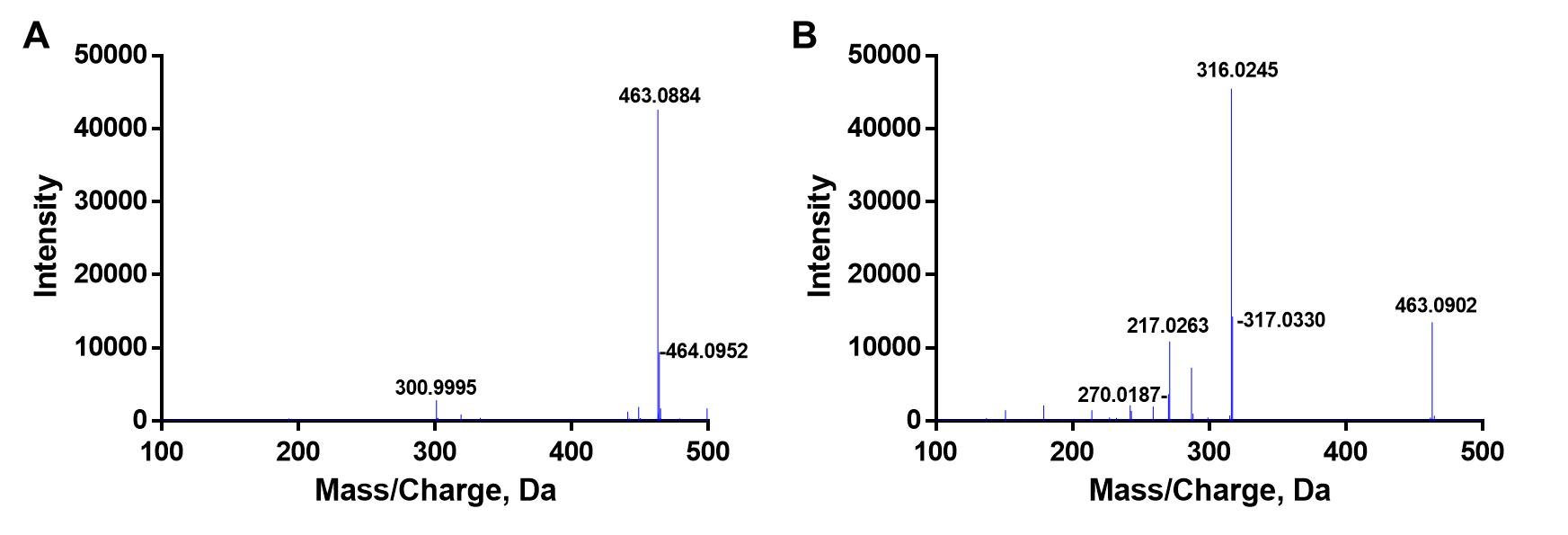


**Fig. S10.** MS^1^ (A) and MS^2^ (B) spectra of myricitrin.


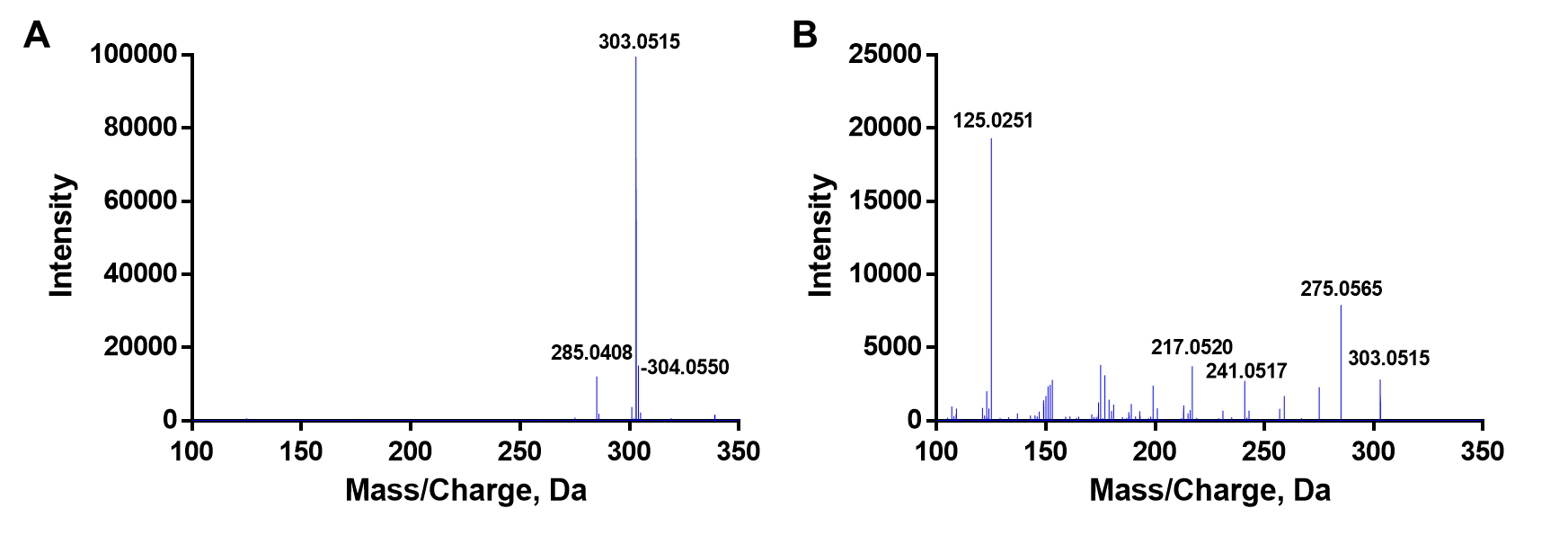


**Fig. S11.** MS^1^ (A) and MS^2^ (B) spectra of taxifolin.


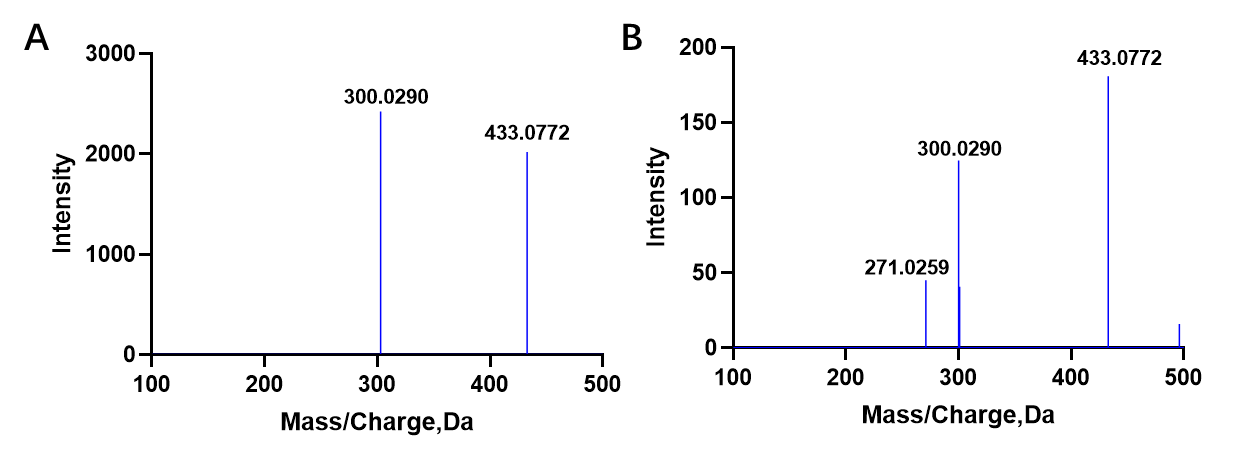


**Fig. S12.** MS^1^ (A) and MS^2^ (B) spectra of reynoutrin.


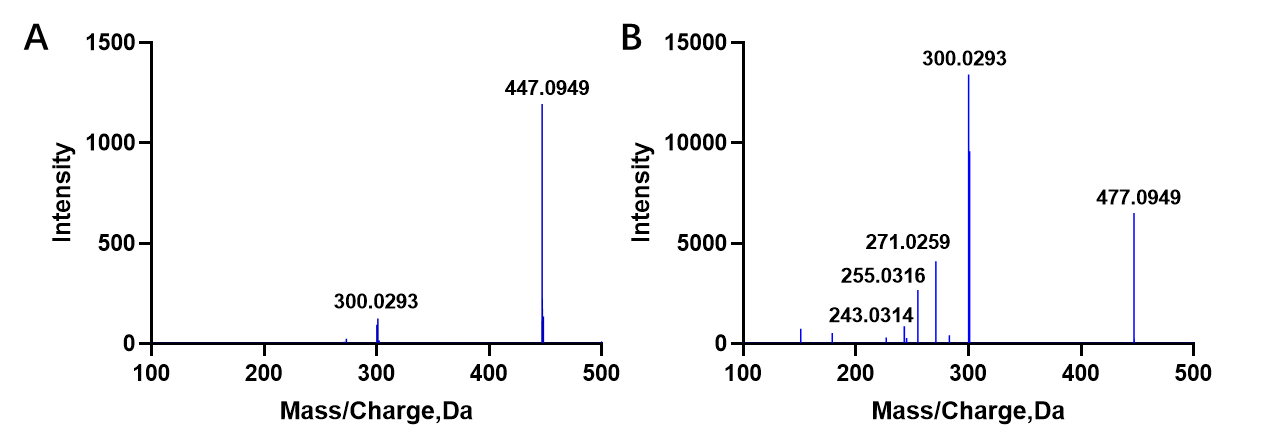


**Fig. S13.** MS^1^ (A) and MS^2^ (B) spectra of quercetin-3-*O*-α-L-rhamnopyranoside.


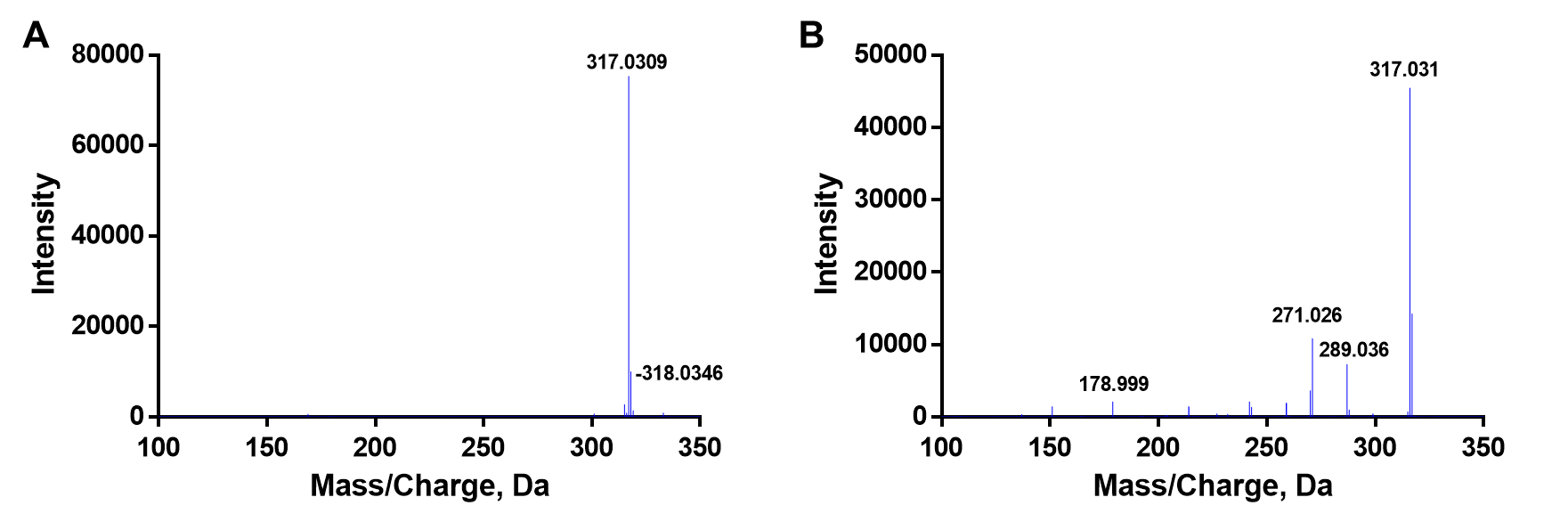


**Fig. S14.** MS^1^ (A) and MS^2^ (B) spectra of myricetin.


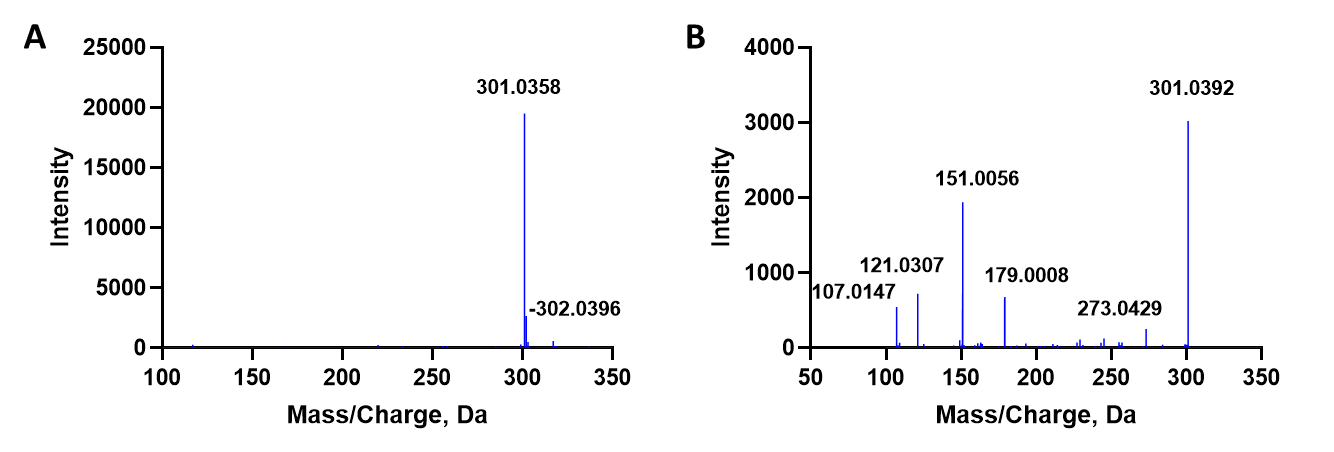


**Fig. S15.** MS^1^ (A) and MS^2^ (B) spectra of quercetin.


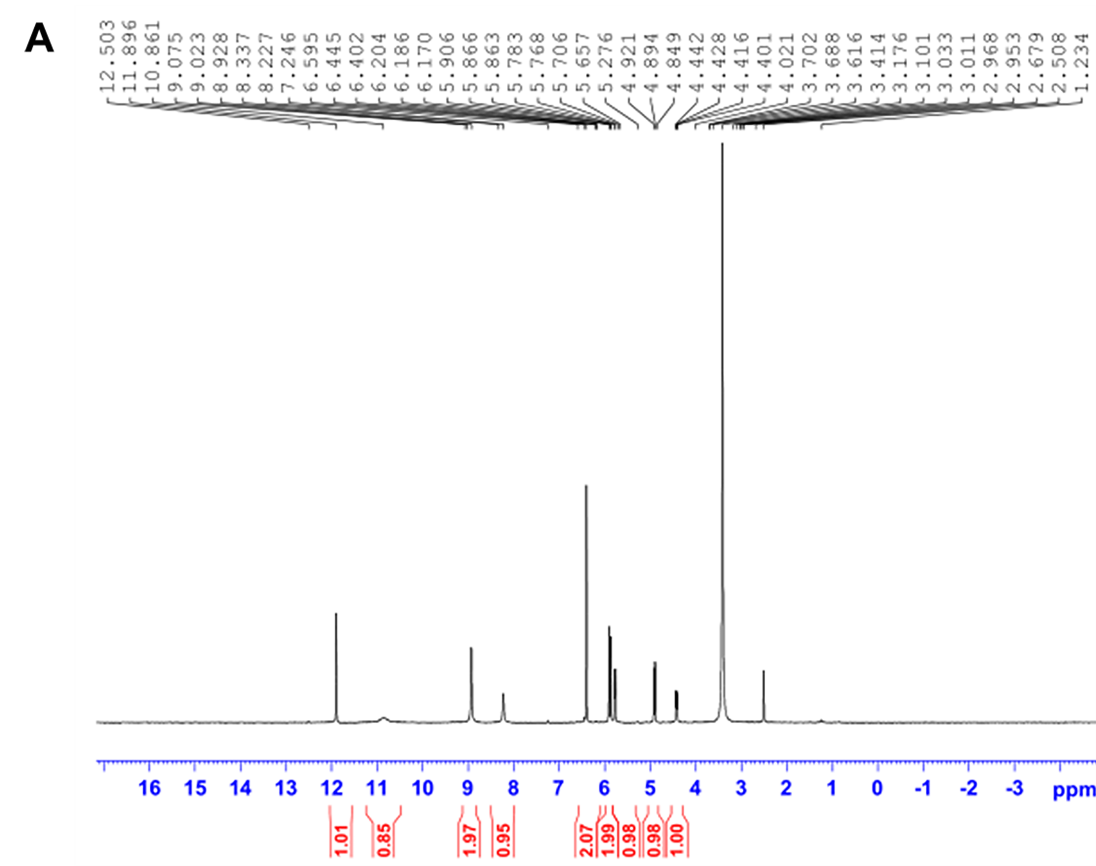

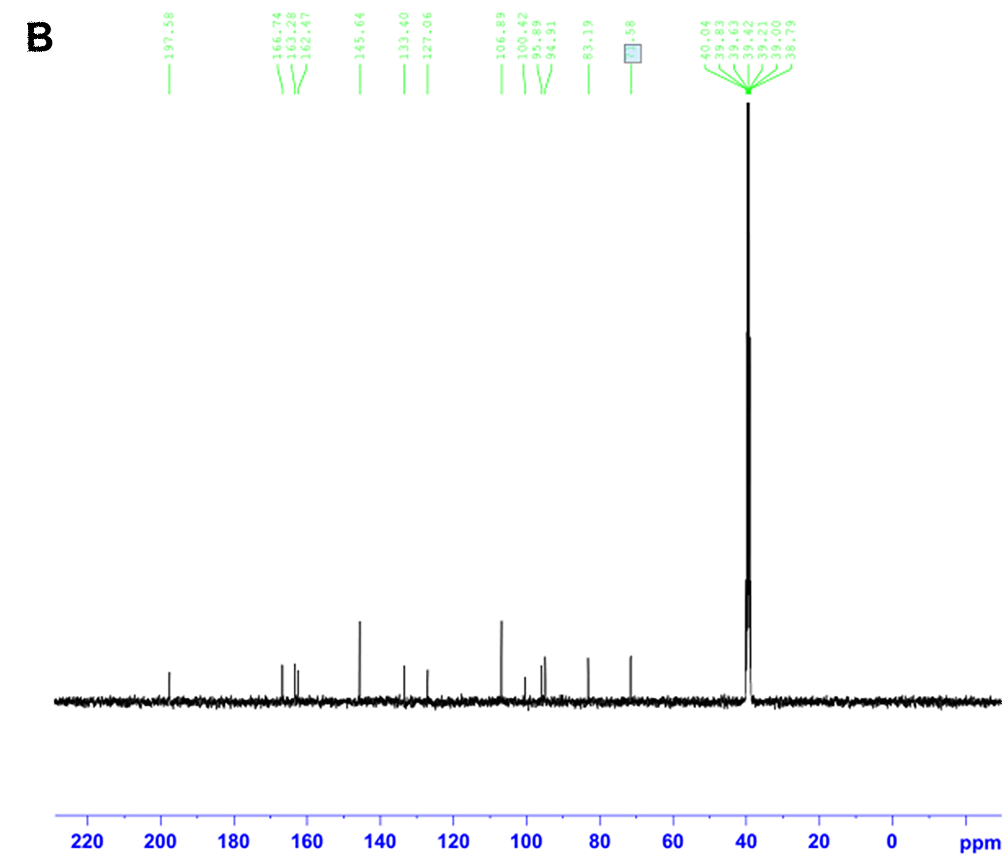


**Fig.S16.** ^1^H NMR (A) and ^13^C NMR (B) spectra of dihydromyricetin.


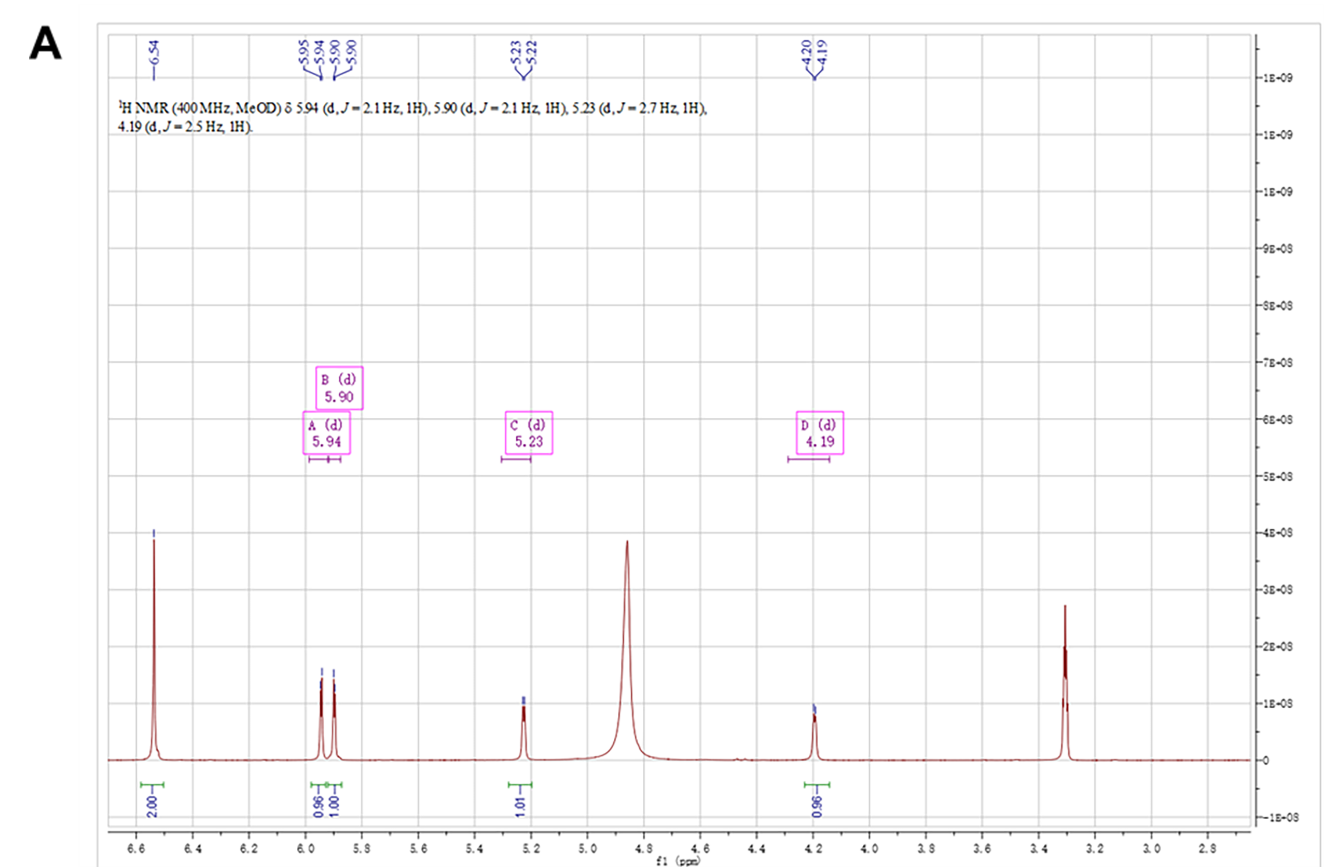

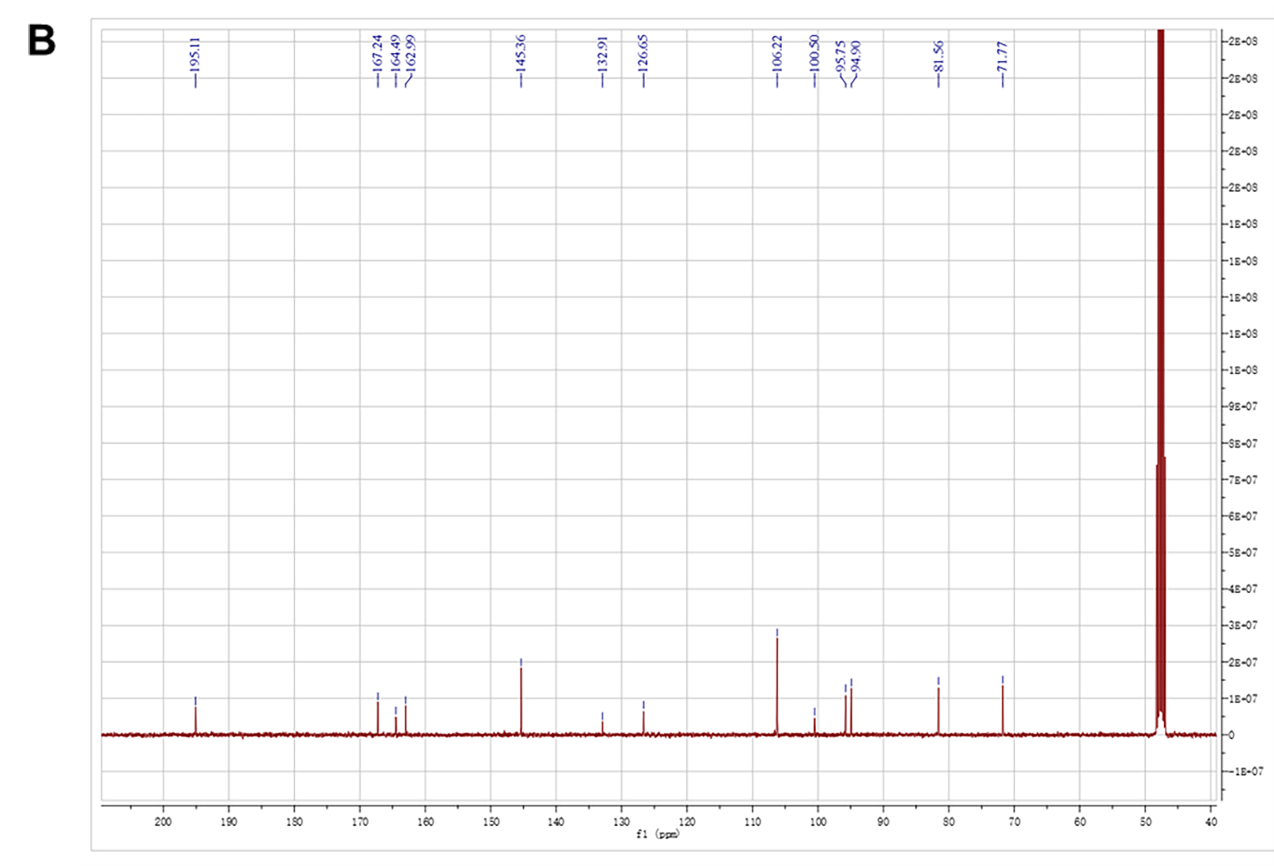

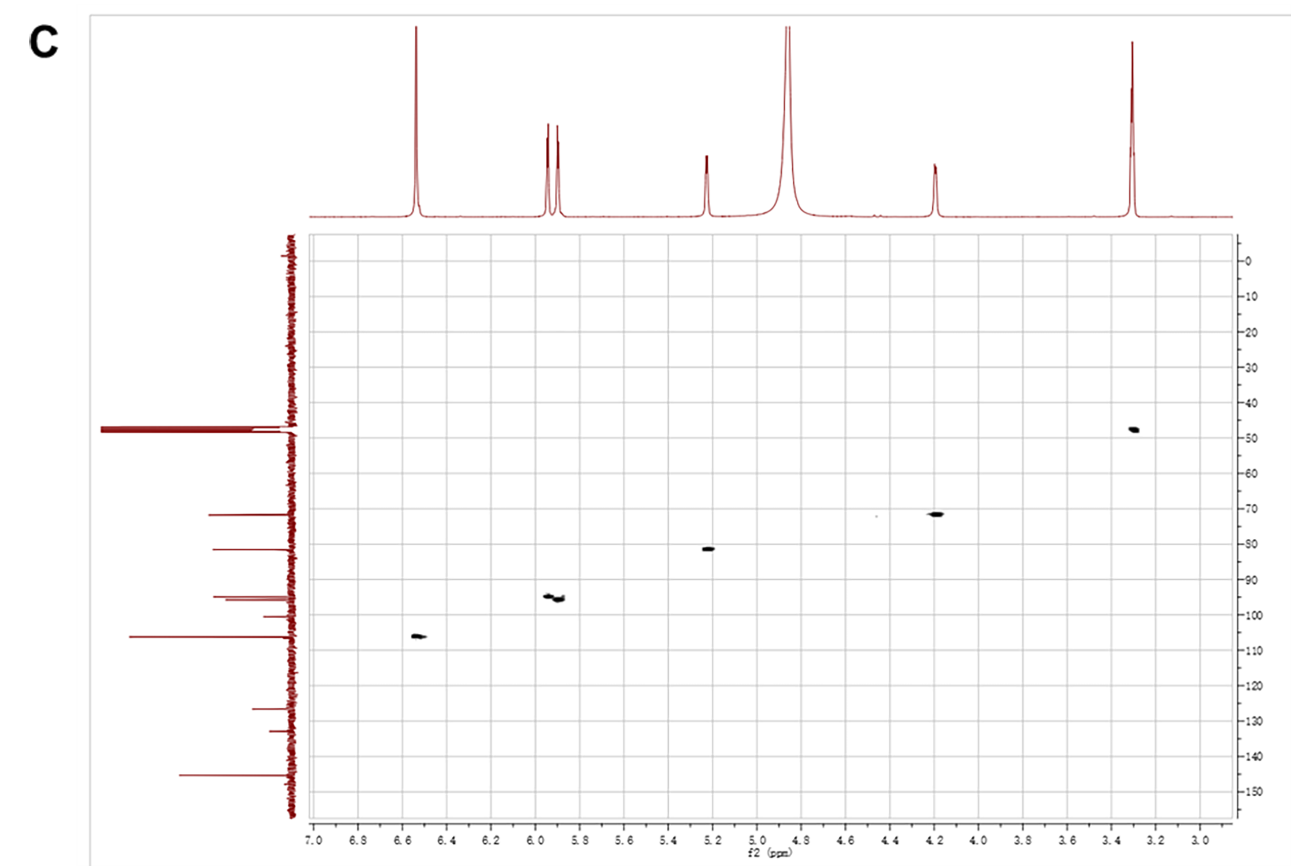

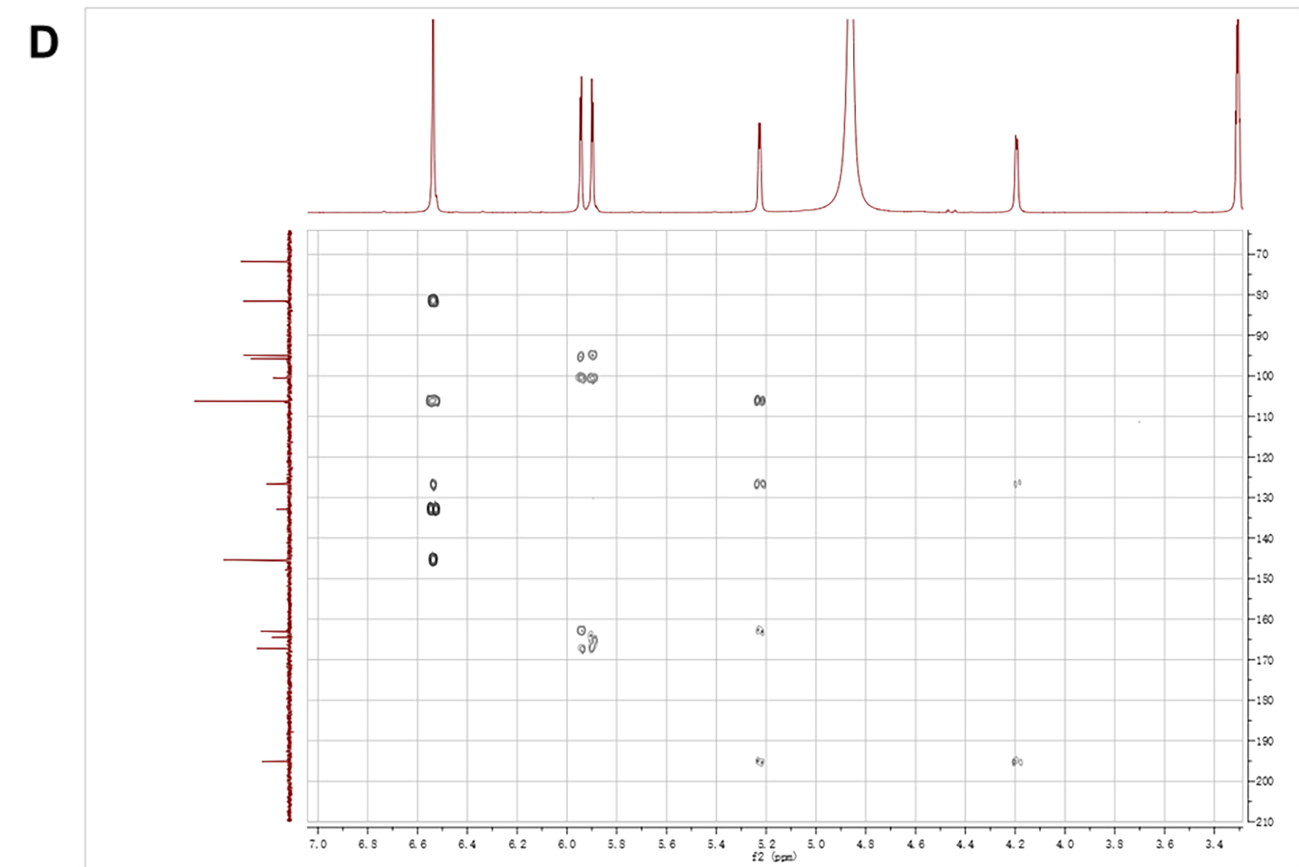


**Fig. S17.** ^1^H NMR (A), ^13^C NMR (B), HSQC(C), and HMBC(D) spectra of iso-dihydromyricetin.


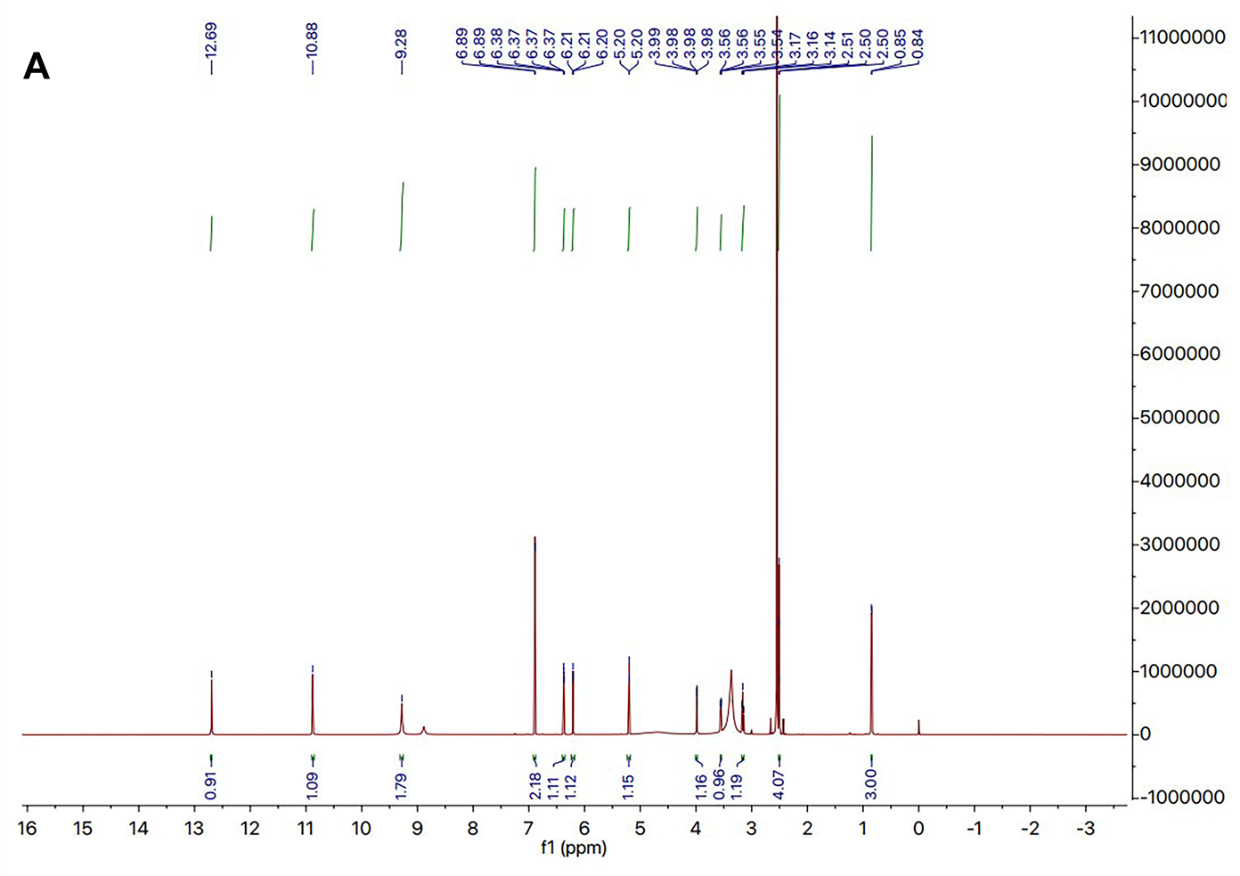


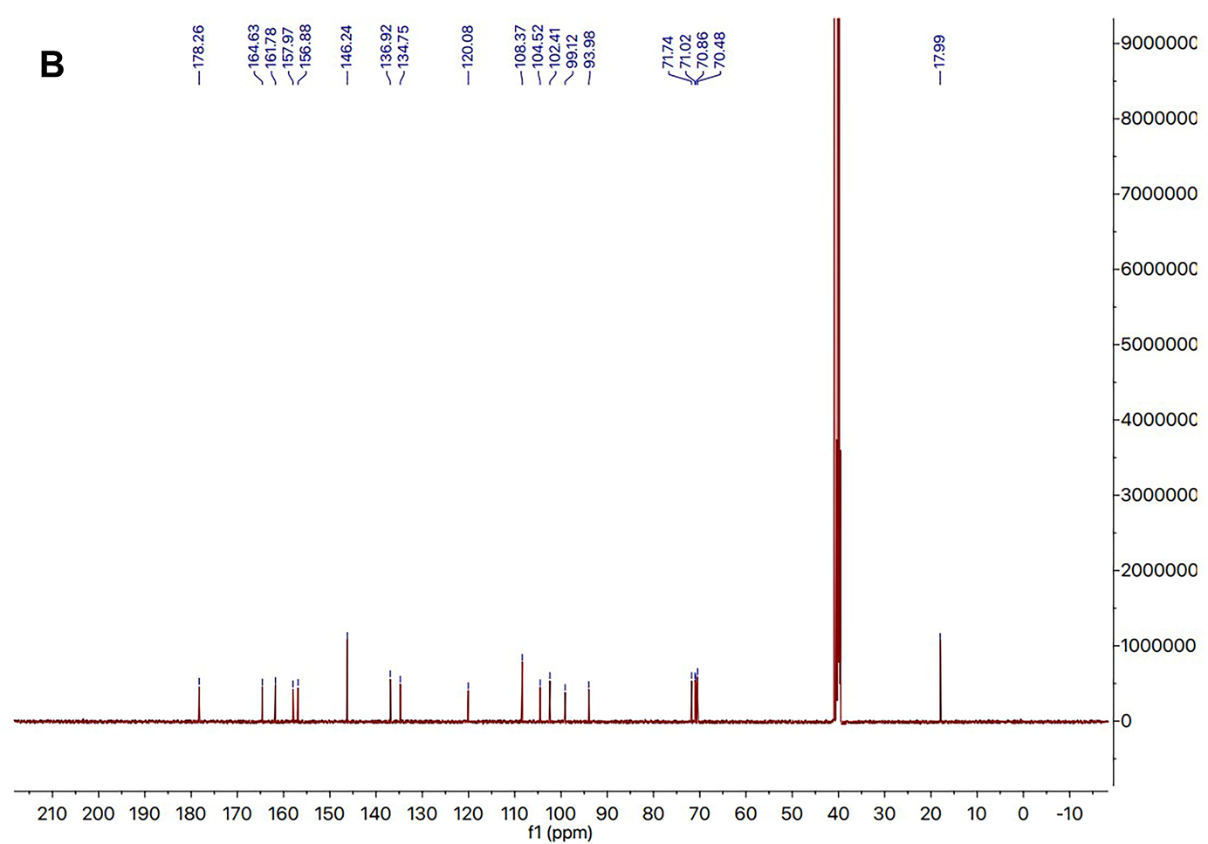


**Fig S18.** ^1^H NMR (A) and ^13^C NMR (B) spectra of myricitrin.


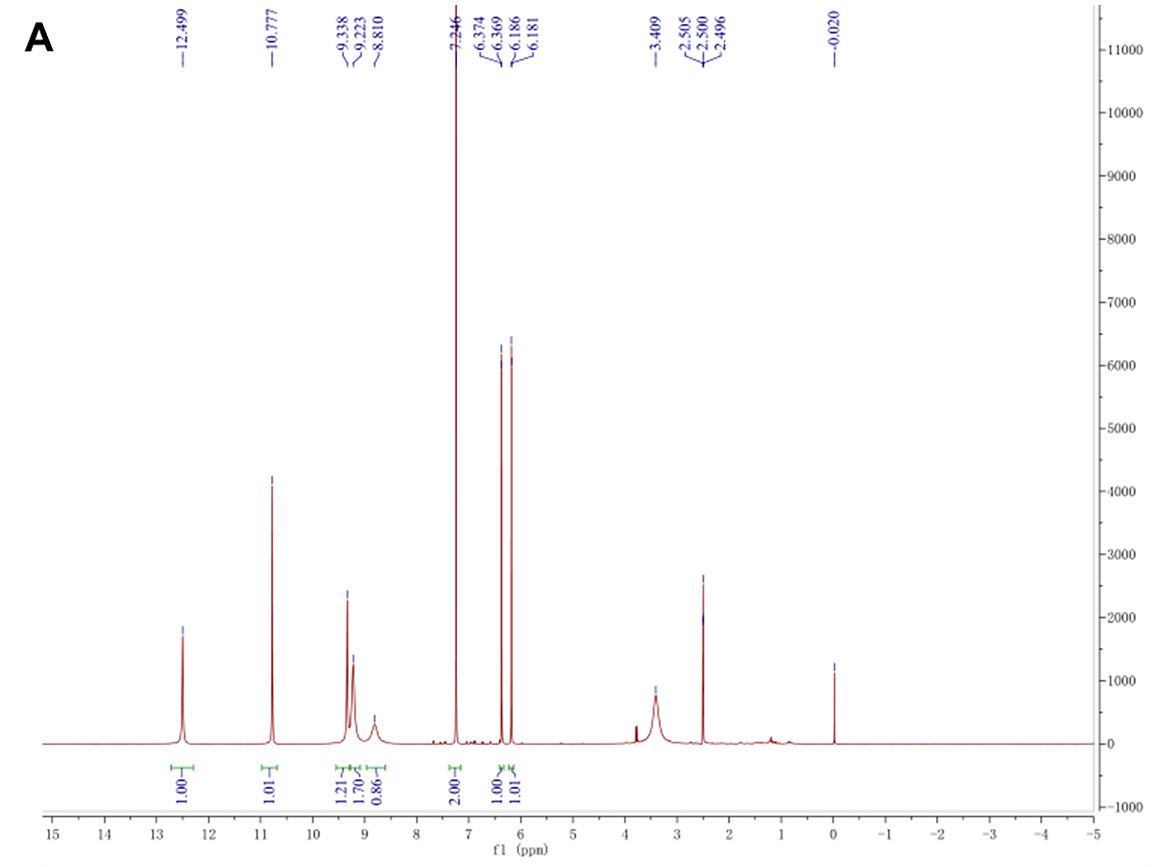

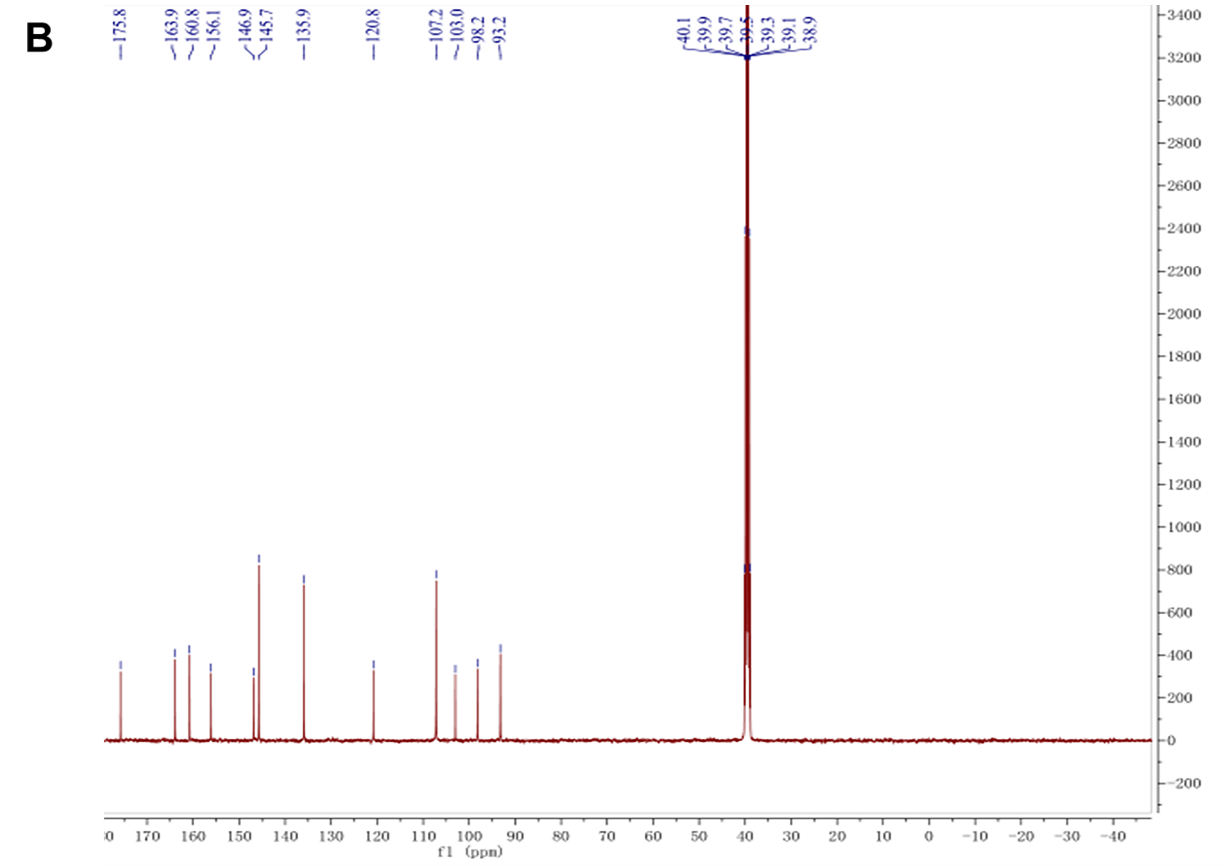


**Fig S19.** ^1^H NMR (A) and ^13^C NMR (B) spectra of myricetin.


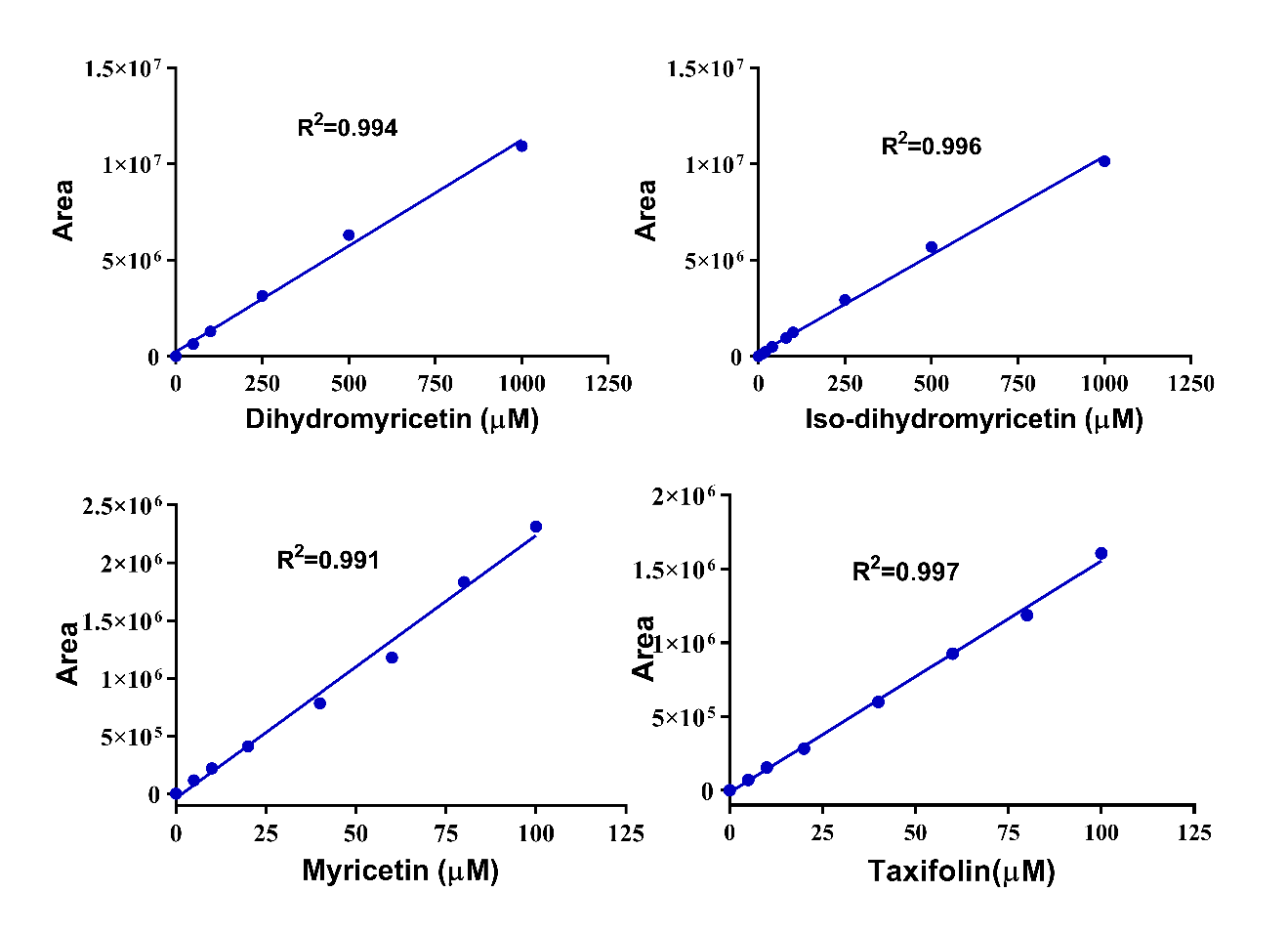


**Fig. S20.** Standard curves for dihydromyricetin, iso-dihydromyricetin, taxifolin, and myricetin were determined.


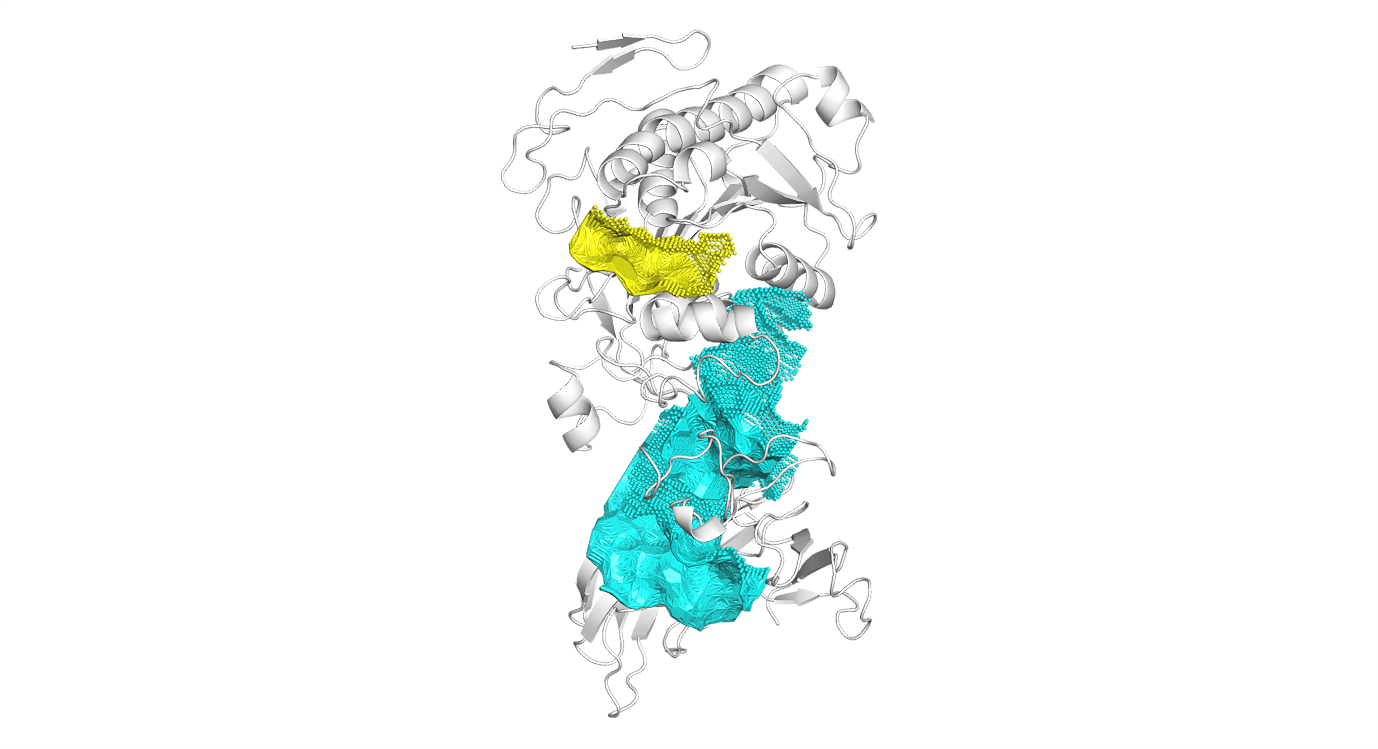


**Fig. S21.** Two druggable pocket predicted by CavityPlus. The yellow pocket was exactly the catalytic site of hPL. The cyan pocket was defined as the allosteric pocket.


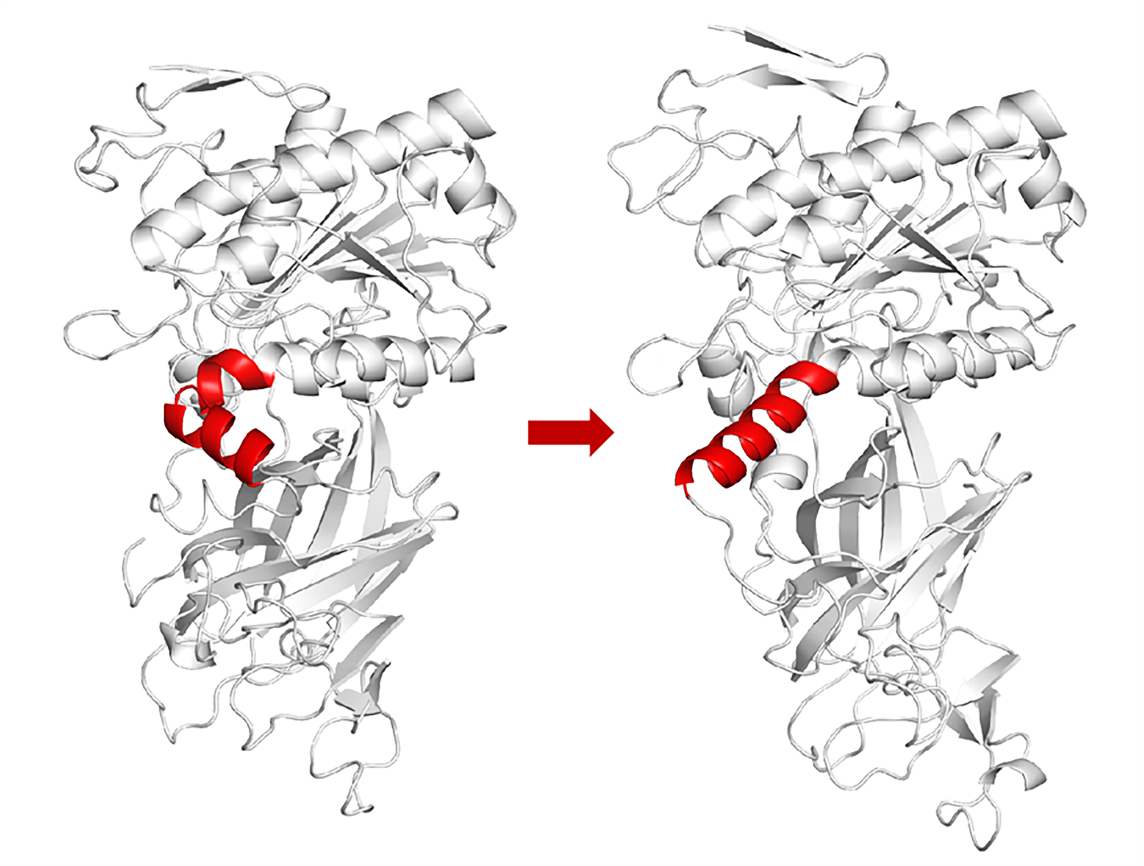


**Fig.S22.** Equilibrium structures of apohPL (left) and myricitrin binding hPL (right). The flap was marked in red.


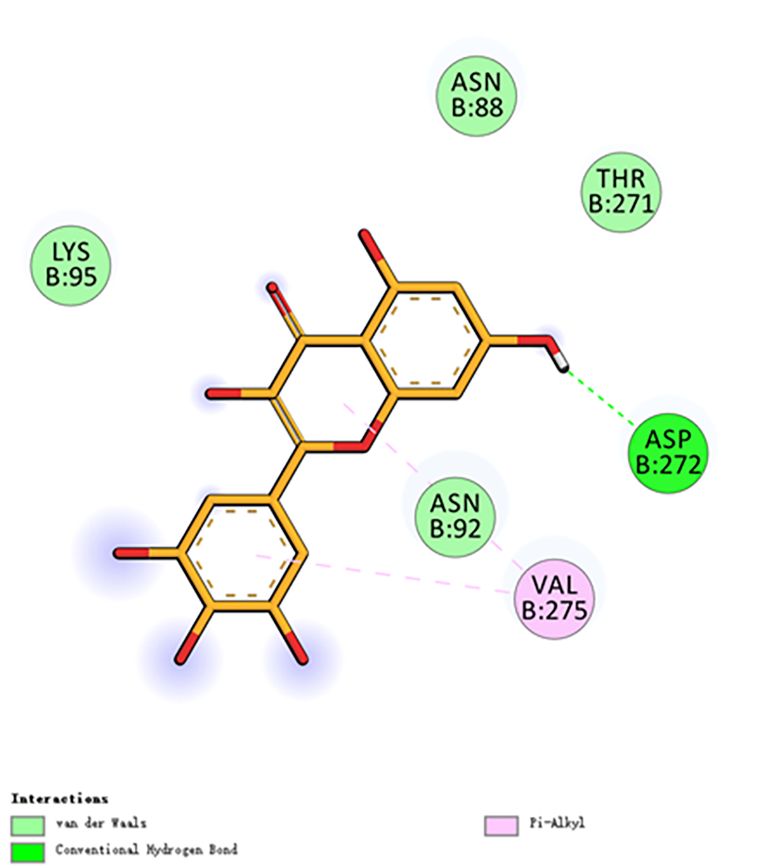


**Fig. S23.** The detailed 2D interactions of myricetin in the surrounding amino acids of the allosteric site of hPL.


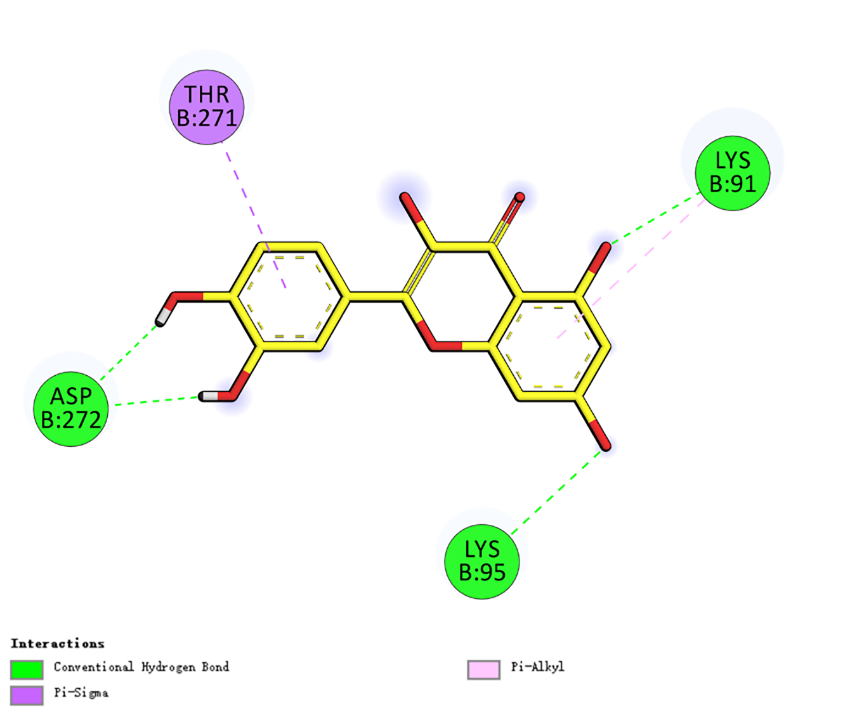


**Fig. S24.** The detailed 2D interactions of quercetin in the surrounding amino acids of the allosteric site of hPL.

**Table S1.** The gradient elution program of mobile phase

| **Time (min)** | **Mobile phase B (acetonitrile)** |
| --- | --- |
| 0-2 | 5 % |
| 2-15 | 30 % |
| 15-20 | 75 % |
| 20-22 | 5 ％ |
| 22-25 | 5 ％ |

**Table S2.** Mass parameters of TOF-MS/MS

| **MS parameters** | **Value/range** | **MS/MS parameters** | **Value/range** |
| --- | --- | --- | --- |
| TOF mass range | 100-2000 | MS/MS mass range | 100-2000 |
| Ion Source Gas 1 | 50 | Declustering Potential | ± 80 |
| Ion Source Gas 2 | 50 | Collision Energy | ± 35 |
| Curtain Gas | 35 | Collision Energy Spread | 20 |
| Ion Spray Voltage Floating （kV） | -4500 / 5500 | Ion Release Delay | 66 |
| on Source Temperature （°C） | 500 | Ion Release Width | 24 |
| Declustering Potential | ± 80 |  |  |
| Collision Energy | ± 10 |  | |
